# Implementation of electronic prospective surveillance models in cancer care: a scoping review

**DOI:** 10.1186/s13012-023-01265-4

**Published:** 2023-04-26

**Authors:** Christian J. Lopez, Kylie Teggart, Mohammed Ahmed, Anita Borhani, Jeffrey Kong, Rouhi Fazelzad, David M. Langelier, Kristin L. Campbell, Tony Reiman, Jonathan Greenland, Jennifer M. Jones, Sarah E. Neil-Sztramko

**Affiliations:** 1 Department of Supportive Care, Princess Margaret Cancer Centre, University Health Network, Toronto, Canada; 2 Institute of Medical Science, University of Toronto, Toronto, Canada; 3 Faculty of Health Sciences, McMaster University, Hamilton, Canada; 4 Faculty of Kinesiology and Physical Education, University of Toronto, Toronto, Canada; 5 Department of Physical Therapy, University of British Columbia, Vancouver, Canada; 6 Library and Information Services, University Health Network, Toronto, Canada; 7 Department of Oncology, Saint John Regional Hospital, Saint John, Canada; 8 Faculty of Medicine, Memorial University of Newfoundland, St John’s, Canada; 9 National Collaborating Centre for Methods and Tools, McMaster University, Hamilton, Canada

**Keywords:** Cancer survivorship, Prospective surveillance model, Patient-reported outcomes, eHealth, Implementation Science, Scoping review

## Abstract

**Background:**

Electronic prospective surveillance models (ePSMs) for cancer rehabilitation include routine monitoring of the development of treatment toxicities and impairments via electronic patient-reported outcomes. Implementing ePSMs to address the knowledge-to-practice gap between the high incidence of impairments and low uptake of rehabilitation services is a top priority in cancer care.

**Methods:**

We conducted a scoping review to understand the state of the evidence concerning the implementation of ePSMs in oncology. Seven electronic databases were searched from inception to February 2021. All articles were screened and extracted by two independent reviewers. Data regarding the implementation strategies, outcomes, and determinants were extracted. The Expert Recommendations for Implementing Change taxonomy and the implementation outcomes taxonomy guided the synthesis of the implementation strategies and outcomes, respectively. The Consolidated Framework for Implementation Research guided the synthesis of determinants based on five domains (intervention characteristics, individual characteristics, inner setting, outer setting, and process).

**Results:**

Of the 5122 records identified, 46 interventions met inclusion criteria. The common implementation strategies employed were “conduct educational meetings,” “distribute educational materials,” “change record systems,” and “intervene with patients to enhance uptake and adherence.” Feasibility and acceptability were the prominent outcomes used to assess implementation. The complexity, relative advantage, design quality, and packaging were major implementation determinants at the intervention level. Knowledge was key at the individual level. At the inner setting level, major determinants were the implementation climate and readiness for implementation. At the outer setting level, meeting the needs of patients was the primary determinant. Engaging various stakeholders was key at the process level.

**Conclusions:**

This review provides a comprehensive summary of what is known concerning the implementation of ePSMs. The results can inform future implementation and evaluation of ePSMs, including planning for key determinants, selecting implementation strategies, and considering outcomes alongside local contextual factors to guide the implementation process.

**Supplementary Information:**

The online version contains supplementary material available at 10.1186/s13012-023-01265-4.

Contributions to the literature

- This scoping review used the ERIC taxonomy, implementation outcomes taxonomy, and the CFIR to advance awareness of the implementation strategies that have been used for ePSMs in oncology, outcomes used to assess ePSM implementation success, and barriers and facilitators to implementation.
- There is a lack of use of implementation science frameworks to understand the approach to implementation of ePSMs in oncology, and use of these frameworks may provide improved guidance for future implementation planning and evaluation.
- The identification of relevant CFIR domains can be used for the theoretically informed development and testing of future strategies to implement ePSMs in oncology.

## Introduction

Cancer is one of the most prevalent, disabling, and costly conditions affecting people worldwide [1, 2]. People with cancer experience deleterious changes to wellbeing including physical, functional, and psychosocial challenges [3, 4]. The presence of cancer-related impairments decreases quality of life and diminishes cancer survivors’ ability to participate in work and life roles meaningfully [5, 6]. Therefore, supportive care strategies to manage treatment-related adverse effects and improve quality of life have become a priority in cancer survivorship research.

Despite the high prevalence of cancer-related impairments, adverse effects of treatments often go undetected and existing interventions to manage these impairments are underutilized [7, 8]. As such, there have been several calls to develop new approaches to care delivery, such as implementing a Prospective Surveillance Model (PSM) into standard care [9, 10]. A PSM includes routine assessment of cancer survivors’ needs and function throughout the cancer care continuum. It may facilitate early identification and intervention to manage anticipated and serious treatment-related adverse effects [9, 10].

Emerging technologies offer a potentially cost-effective and patient-centered solution to implement a PSM into clinical practice. An electronic PSM (ePSM) includes remote monitoring of patients at specified time points throughout their care using electronic patient-reported outcomes (ePROs) [9, 10]. ePROs provide a direct measurement of patient experiences and have been shown to be feasible and provide a reliable estimate of patients’ health and needs [11, 12]. An ePSM may also include an automated triage system to provide education and self-management materials and assist the oncology team with the assessment and synthesis of patient data to improve patient-provider conversations and help clinicians make appropriate referrals. Therefore, an ePSM has the potential to provide timely access to information and services to manage treatment-related symptoms and reduce rates of disability and dysfunction [9, 13].

While randomized controlled trials have demonstrated that ePSMs are effective at improving quality of life and decreasing symptom distress and emergency room visits, as well as associated with increased survival [14, 15], less is known about the implementation of ePSMs into routine care. Known barriers to implementation include a lack of resources for designing the system, ambiguity around appropriate risk stratification criteria to guide referral pathways, and time constraints for providers to address needs that arise from ePRO scores [11, 12, 16].

Using an implementation science approach to move evidence-based practices such as an ePSM into routine clinical care has been identified as a priority for future research in cancer survivorship [17]. A comprehensive summary of the reported barriers and facilitators to implementing ePSMs, as well as the implementation strategies and corresponding outcomes that have been utilized, is necessary to facilitate ePSM use in routine cancer care. This scoping review aimed to provide a comprehensive synthesis of the approach to implementation reported in studies evaluating the use of ePSMs in oncology.

## Methods

We conducted a scoping review following guidance by the Joanna Briggs Institute [18] and the Preferred Reporting Items for Systematic Reviews and Meta Analyses Scoping Review reporting recommendations (Additional file 1). The following research questions guided this review:

1. What theories, models, and frameworks (TMFs) have been used to guide the implementation planning and evaluation of ePSMs in oncology?
2. What implementation strategies have been used to promote the implementation of an ePSM in oncology?
3. What outcomes have been used to assess the success of the implementation of ePSMs in oncology?
4. What is known about the determinants (barriers and facilitators) to the implementation of ePSMs in oncology?

### Data sources and search strategy

A search was performed in Medline ALL (Medline and Epub Ahead of Prints and In-Process, In-Data-Review & Other Non-Indexed Citations), Embase Classic + Embase, Cochrane Central Register of Controlled Trials, Cochrane Database of Systematic Reviews, Emcare, and PsycInfo (all from the OvidSP platform), and CINAHL from EBSCOhost from inception to February 2021. Each search strategy comprised a combination of controlled vocabulary and text words, adapting the database-specific search syntax. The search was restricted to human studies and adults over 18, excluding books and conferences. There were no language restrictions (see Additional file 2 for all search strategies). Reference lists of relevant reviews and included studies were hand searched, and authors of relevant conference abstracts were contacted for full texts.

### Study eligibility criteria

Eligible studies described the real-world implementation of an ePSM for adult cancer survivors (age 18 years and older). For this review, an ePSM must have included the routine collection of ePROs as surveillance to monitor and act on patients’ responses. “Routine” was defined as the systematic use of outcome measure(s) in clinical practice with every eligible patient as part of a standardized assessment [19], as previously reported [16]. Given that the objective of this review was to identify the existing data related to implementation to inform future implementation efforts, we included articles reporting on studies that (1) explicitly used implementation science in their design, data collection, and analysis; or (2) studies that reported on the implementation of an ePSM for routine care but did not use an implementation science approach. The latter were included because while these studies may not have used implementation science explicitly, the approaches used to facilitate implementation (i.e., strategies), outcomes collected, and barriers and facilitators reported provided relevant data that could be used to inform future approaches to implementation. However, studies reporting on the preliminary development of an ePSM (e.g., proof-of-concept) were excluded. Studies that focused on routine collection of ePROs which did not include the option to act on patients’ responses (e.g., establishment of a longitudinal cohort or research database) were excluded. Experimental, observational, qualitative, and mixed methods studies were included, while opinion pieces, guidelines, and published conference abstracts were excluded.

### Study selection

After duplicates were removed, identified citations were exported to Covidence systematic review software. Two reviewers independently screened each title and abstract in duplicate. The full texts of all potentially eligible articles were retrieved and assessed independently by two independent reviewers. Disagreements were resolved through discussion during bi-weekly meetings.

### Data extraction

Relevant study information was extracted, including ePSM system characteristics and implementation details (e.g., TMFs, implementation strategies, outcomes, and barriers and facilitators). Two reviewers extracted data from all studies independently and in duplicate, and disagreements were resolved through discussion during bi-weekly meetings.

### Data synthesis

A descriptive analysis was used to summarize the characteristics of the included studies, the TMFs utilized, implementation strategies used, the outcomes measured, and barriers and facilitators reported. Articles reporting on the same implementation project were analyzed as a single ePSM intervention; however, these studies were reported separately when the same ePSM system was adapted and implemented in different populations or settings. This decision was made as these studies may have used different implementation strategies, assessed different outcomes, or reported different determinants.

Before data analysis, all coded data on TMFs, strategies, outcomes, and determinants were reviewed by two independent reviewers, and disagreements were resolved through discussion. TMFs were categorized as (1) classic theories, which originate from fields outside of implementation science; (2) implementation theories, which implementation researchers have developed; (3) process models, which describe and/or guide the process of translating research into practice; (4) determinant frameworks, which describe factors that may impact implementation; and (5) evaluation frameworks, which specify aspects of implementation that could be evaluated to determine implementation success [20].

The Expert Recommendations for Implementing Change (ERIC) taxonomy [21] was used to label the implementation strategies described by the included articles. Team members extracted the specific terminology used to describe strategies in each study and coded the strategy based on definitions provided by the ERIC project. Each study was coded into one or more of 73 discrete implementation strategies which belong to one of nine thematic clusters, including (1) the use of evaluative and iterative strategies, (2) providing interactive assistance, (3) adapting and tailoring to the context, (4) developing stakeholder interrelationships, (5) training and educating stakeholders, (6) supporting clinicians, (7) engaging consumers, (8) utilizing financial strategies, and (9) changing the infrastructure [22]. For data coding, the definitions from the original ERIC list were slightly adapted for an ePSM intervention (see Additional file 3). For example, changing record systems involved integrating the ePSM into the electronic medical record or a patient portal. Intervening with patients to enhance adherence and uptake involved using system alerts to patients based on inactivity or using in-person reminders to complete ePROs when patients attend a clinic visit. Lastly, changing equipment encompassed setting up computer stations or obtaining tablets for the clinic for patients to complete their screening questions.

Proctor’s implementation outcomes taxonomy was used to categorize the outcomes used to assess implementation, including (1) acceptability, (2) adoption, (3) appropriateness, (4) feasibility, (5) fidelity, (6) cost, (7) reach/penetration, and (8) sustainability [23], following guidance for the use of these outcomes for projects using patient-reported outcomes by Stover and colleagues [24]. The definitions from the implementation outcomes taxonomy were adapted for an ePSM intervention to facilitate the categorization (see Additional file 4). This provided specific measures for evaluating the implementation of an ePSM that could be used to resolve discrepancies between the terminology utilized by the included studies and the implementation outcomes taxonomy. For instance, while studies may report on the feasibility of an ePSM by assessing ePRO completion rates, Stover et al. [24] categorized this measure as fidelity. Similarly, while studies may report on the acceptability of an ePSM by assessing perceptions regarding the fit of the system with the patient population, Stover et al. [24] categorized this measure as appropriateness.

Reported barriers and facilitators to implementation were analyzed according to the Consolidated Framework for Implementation Research (CFIR) [25], a widely used determinant framework that includes 39 constructs within five domains (characteristics of the intervention, inner setting, outer setting, characteristics of individuals, and the process of implementation). The CFIR codebook template [26], which provides descriptions for each construct, guided the classification of the barriers and facilitators. First, one author (CL) categorized the barriers and facilitators extracted based on the five CFIR domains, and then coded the data according to the CFIR constructs within each domain. A second coder (KT) reviewed all the coded data and both coders met to discuss any necessary refinements.

## Results

**Fig. 1:**
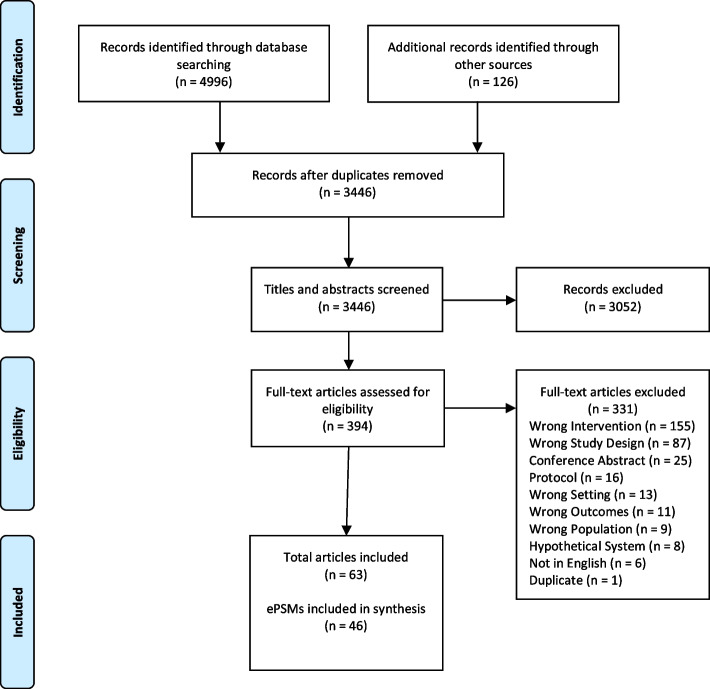
Preferred Reporting Items for Systematic Reviews and Meta-Analyses flow diagram

The database search yielded 4996 records, and 126 records were identified through reference checking of included articles and relevant reviews (Fig. 1). Following the removal of duplicates, 3446 citations underwent title and abstract screening, and 394 full-text articles were reviewed. Of these, 63 articles describing 46 interventions met all inclusion criteria (Table 1). While publication years ranged from 2005 to 2021, the majority were published in the last 5 years (*n* = 43, 68%). Of the 46 interventions included, nearly half (*n* = 22, 48%) were conducted in Europe [27–48], followed by North America (*n* = 20, 43%) [14, 15, 49–66], Australia (*n* = 3, 7%) [67–69], and the Philippines (*n* = 1, 2%) [70]. Most interventions targeted patients with a mix of cancer types (*n* = 24, 52%) [14, 15, 27, 30, 35, 37, 40, 41, 46, 47, 51, 52, 54, 55, 60–63, 65–70], followed by a focus on head and neck (*n* = 4, 8%) [31, 32, 48, 53], gynecologic (*n* = 3, 7%) [49, 57, 58], lung (*n* = 3, 7%) [39, 56, 59], and breast (*n* = 3, 7%) [28, 42, 64] cancers. Of the 46 ePSM studies, 33% (*n* = 15) explicitly used implementation science in their design, data collection, or analysis [30, 32, 35, 41, 44, 46, 50, 51, 53–55, 58, 60, 67, 69], while 67% (*n* = 31) reported on the implementation of an ePSM but did not use an implementation science approach [14, 15, 27–29, 31, 33, 34, 37–40, 42, 43, 47–49, 52, 56, 57, 59, 61–66, 68, 70–72]. Overall, 57% (*n* = 26) used a non-randomized experimental or quality improvement design [29, 31–34, 37–39, 41, 43, 44, 47–50, 52, 55–59, 62, 64, 65, 67, 68], 26% (*n* = 12) used a randomized experimental design [14, 15, 27, 28, 35, 40, 42, 46, 54, 70, 71, 73], 9% (*n* = 4) were descriptive case reports on the implementation of the intervention [53, 60, 61, 63], 4% (*n* = 2) used an observational design [30, 74], and 4% (*n* = 2) solely used a qualitative design [36, 66]. Notably, 30% (*n* = 14) of the studies included an additional qualitative component to their design [29, 31, 32, 35, 38, 39, 42, 45, 48, 50, 51, 67–69]. Within included studies, 41% (*n* = 19) focused exclusively on patients on active treatment [14, 15, 27, 29, 37, 40, 41, 44, 45, 47, 50, 54–57, 61, 62, 64, 65], 39% (*n* = 18) included patients during active treatment as well as follow-up surveillance [28, 30, 31, 33, 34, 38, 39, 43, 51–53, 58, 60, 63, 66–69], 11% (*n* = 5) were exclusively during follow-up surveillance [32, 36, 42, 46, 48], 4% (*n* = 2) during the postoperative period [49, 59], and 4% (*n* = 2) during palliative care [35, 70].

**Table 1:** Characteristics of included studies

Author	System name	Setting	Intervention design	Cancer type	Treatment phase	Symptoms and ePROs collected	Surveillance frequency and duration	ePSM patient features	ePSM provider features
Abernethy 2009 [64]	PACE system and Patient Care Monitor	USA, outpatient clinic located within a university medical center	Single-arm intervention	Breast	Active treatment	Treatment-related symptoms; psychological, functional, and social concerns; FACT-G, FACT-B, MDASI, FACIT-F	Patient’s 4 consecutive scheduled clinic visits for 6 months	Self-management	Summary reports
Absolom 2021 [27, 75, 76]	eRAPID	UK, outpatient clinics located within a cancer center	RCT	Breast, colorectal, and gynecologic	Active treatment	CTCAE	Weekly for 18 weeks	Self-management, score history, and system message	Summary reports and alerts
Bacorro 2018 [70]	iComPAsS	Philippines	RCT	Any	Palliative care	ESAS	Set by the physician based on pain severity for 20 weeks	Communication and circle of care	Summary reports, communication, and patient list
Baeksted 2017 [29]	AmbuFlex software	Denmark, outpatient clinic located within a university hospital	Single-arm intervention + qualitative interviews	Prostate	Active treatment	CTCAE	Every 3 weeks (duration not specified)	NR	Summary reports
Basch 2005 [57]	STAR	USA, outpatient clinic located within a cancer center	Single-arm intervention	Gynecologic	Active treatment	PRO-CTCAE, ECOG, EQ-5D	Every outpatient visit for 8 weeks	System message	Alerts and summary reports
Basch 2007 [56]	STAR	USA, outpatient clinic located within a cancer center	Single-arm intervention	Lung	Active treatment	PRO-CTCAE, Karnofsky Performance Status, EQ-5D	Every outpatient visit for 16 months, or until death, voluntary withdrawal, or transfer of care	System message	Summary reports
Basch 2016 [14]	STAR	USA, outpatient clinic located within a cancer center	RCT	Breast, gynecologic, genitourinary and lung	Active treatment	CTCAE, ECOG, EQ-5D	Any medical oncology visit or infusion suite visit until treatment completion	System message	Alerts and summary reports
Basch 2020 [54]	PRO-TECT	USA, community oncology practices	Cluster RCT	Any	Active treatment	CTCAE, ECOG, FT12 from the COST-FACIT	Weekly for 12 months or until treatment completion	Self-management	Alerts, summary reports, and recommended actions
Berry 2014 [15, 77]	ESRA-C	USA, outpatient clinics located within two cancer centers	RCT	Any	Active treatment	Symptom distress scale	Four assessments including before starting a new treatment, 3–6 weeks and 6–8 weeks later, and 2–4 weeks after treatment completion	Self-management and score history	Summary reports
Biran 2020 [50]	Medocity Home Health	USA, outpatient clinic located within a medical center	Single-arm intervention + qualitative focus groups and interviews	Multiple myeloma	Active treatment	CTCAE	Weekly for 4 weeks	Self-management and system message	Alerts and summary reports
Børøsund 2014 [28]	WebChoice	Norway, outpatient clinics located within university and regional hospitals	RCT	Breast	Active treatment and follow-up	Physical, functional, and psychosocial symptoms and concerns	Patients could use the system on a voluntary basis for 6 months	Self-management, peer support, and general education	NR
Cowan 2016 [49, 78]	STAR	USA, cancer centre	Single-arm intervention	Gynecologic	Post-op	CTCAE, EORTC QLQ-C30	Weekly for 6 weeks	System message	Alerts and summary reports
de Wit 2019 [30]	OncoKompas	Netherlands, hospitals	Cross-sectional	Any	Any	Quality-of-life domains including physical, psychological, and social functioning, healthy lifestyle, and existential issues	Every outpatient visit (duration not specified)	Self-management and score history	NR
Dronkers 2020 [31]	Healthcare Monitor	Netherlands, outpatient clinic located within a university medical center	Single-arm intervention + qualitative interviews	Head and neck	Any	HADS, EAT10 Voice Handicap Index, EQ5LD-3L, and versions of the EORTC	Every outpatient visit for up to 5 years post treatment	NR	Summary reports
Dudgeon 2012 [63, 79, 80]	NR	Canada, outpatient clinics located within regional cancer centers	Case report	Any	Any	ESAS	Every outpatient visit (duration not specified)	NR	Summary reports and recommended actions
Duman-Lubberding 2017 [32]	OncoQuest	Netherlands, outpatient clinic located within a university medical center	Single-arm intervention + qualitative interviews	Head and neck	Follow-up	EORTC QLQ-C30, QLQ-H&N35, HADS	Every outpatient visit (duration not specified)	NR	Summary reports
Erharter 2010 [33]	CHES software	Austria, outpatient clinic located within a medical university	Single-arm intervention	Brain	Active treatment and follow-up	EORTC QLQ-C30, QLQ-BN20	Every outpatient visit (duration not specified)	NR	Summary reports
Fernandez 2019 [34]	ORION software	UK, outpatient clinics within two tertiary centers	Single-arm intervention	Brain	Any	EORTC QLQ-C30 and QLQ-BN20	Every outpatient visit (duration not specified)	NR	Summary reports
Garcia 2019 [52]	Assessment Center software	USA, outpatient clinics located within a cancer center	Single-arm intervention	Any	Any	PROMIS, Distress Thermometer, Patient-Generated Subjective Global Assessment	Every outpatient visit (duration not specified)	System message	Alerts and summary reports
Girgis 2017 [68, 81]	PROMPT-Care	Australia, cancer centers of public hospitals	Single-arm intervention + qualitative semi-structured interviews	Any solid tumor	Any	Distress Thermometer, ESAS, SCNS-ST9	Every 2 weeks for patients receiving treatment and monthly for those who were in follow-up (duration not specified)	Self-management	Summary reports
Girgis 2020 [67, 82]	PROMPT-Care 2.0	Australia, cancer centers of public hospitals	Propensity matched study + qualitative semi-structured interviews	Any solid tumor	Any	Distress Thermometer, ESAS, and SCNS-ST9	Monthly until voluntary withdrawal or death	Self-management	Alerts, summary reports, and recommended actions
Hackett 2020 [35, 83]	PainCheck	UK, outpatient oncology and palliative care clinics	RCT + qualitative semi-structured interviews	Any	Palliative care	BPI and Coping Strategies Questionnaire	Daily for 14 weeks (up to 14 days after their 12-week follow-up assessment)	Self-management, score history, and system message	Alerts, summary reports, and communication
Thestrup Hansen 2021 [36, 72]	NR	Denmark, hematological outpatient clinic at a university hospital	Qualitative ethnography	Hematologic	Follow-up	EORTC QLQ-C30 and OEQ	Every outpatient visit for 2 years	NR	Summary reports
Hauth 2019 [37]	PROMetheus	Germany, outpatient oncology clinic	Single-arm intervention	Any	Active treatment	CTCAE	Weekly (duration not specified)	NR	Summary reports
Howell 2020 [51, 74]	NR	Canada, outpatient oncology clinics located within regional cancer centers	Observational cohort + qualitative focus groups	Any	Any	ESASr, BPI, Cancer Fatigue Scale, GAD-7, SDI-21, PHQ-9, and quality of life (1-item)	Every outpatient visit (duration not specified)	NR	Summary reports
Kneuertz 2020 [59]	SeamlessMD App	USA	Single-arm intervention	Lung	Post-op	Pain, anxiety, and mood	Daily for 1 month	Self-management, score history, communication, and general education	Summary reports
Li 2016 [60]	DART	Canada, outpatient clinics located within a cancer center	Descriptive case report	Any	Any	ESAS-r, ECOG, PHQ-9, GAD-7, SDI-21, Canadian Problem Checklist	Every outpatient visit (duration not specified)	NR	Summary reports
Maguire 2015 [39]	ASyMS	UK	Single-arm intervention + qualitative semi-structured interviews	Lung	Active treatment and follow-up	Memorial Symptom Assessment Scale-Short Form, Rotterdam Symptom Checklist-Activity Subscale	Daily for up to 1 month after treatment completion	Self-management	Alerts and summary reports
Maguire 2020 [38]	AsyMSmeso	UK	Single-arm intervention + qualitative focus groups and semi-structured interviews	Malignant pleural mesothelioma	Any	Symptoms include breathlessness, pain, fatigue, appetite, constipation, cough, and sweating	Daily for 2 months	Self-management and score history	Alerts and summary reports
Maguire 2021 [40, 84, 85]	ASyMS	Austria, Greece, Norway, Ireland, and UK cancer centers	RCT	Breast, colorectal, and lymphoma	Active treatment	CTAQ	Daily for up to a maximum of 6 cycles of chemotherapy	Self-management, score history, and circle of care	Alerts, summary reports, recommended actions
Mark 2008 [66]	PACE system and Patient Care Monitor	USA, outpatient community oncology clinics	Qualitative study	Any	Active treatment and follow-up	General physical symptoms, treatment side effects, acute distress, despair, impaired ambulation, impaired performance, and quality of life	Every outpatient visit (duration not specified)	General education	Summary reports
Mouillet 2021 [41]	REMOQOL and CHES software	France, university hospital	Single-arm intervention	Breast, colorectal, and lung	Active treatment	Q-5D 3L, and cancer-specific versions of the EORTC	Every outpatient visit for 4 months	NR	Summary reports
Naughton 2020 [55]	NR	USA, outpatient oncology clinics located within a university cancer center	Single-arm intervention	Gynecologic and breast	Active treatment	Includes pain, fatigue, sleep quality, quality of life, and PHQ9	Monthly for 12 months or until the end of active therapy, entry into hospice, or when the patient or physician requests to stop the surveys	NR	Alerts
Riis 2021 [42]	SurveyXact software	Denmark, outpatient oncology clinic located within a university hospital	RCT + qualitative focus groups	Breast	Follow-up	EORTC QLQ C30 and QLQ-BR23	Quarterly for 2 years	Communication	Summary reports
Roberts 2020 [69, 73]	iPROMOS	Australia, outpatient oncology clinics located within a tertiary teaching/ quaternary referral hospital	Cluster stepped-wedge trial + qualitative field notes, case report forms, memos, and survey questions	Any	Active treatment and follow-up	CTCAE	Every outpatient visit (duration not specified)	Score history	Summary reports
Rotenstein 2017 [61]	Tonic software	USA, outpatient oncology clinics	Descriptive case report	Gastrointestinal, gynecologic, genitourinary, breast, brain, and head and neck	Active treatment	FACT-G, PROMIS-10, Bladder Cancer Index, EPIC-26, and disease specific versions of the FACT, EORTC, and MDASI	Every outpatient visit (duration not specified)	NR	Summary reports
Strachna 2021 [53]	Head and Neck PROs Oncology Platform	USA, outpatient oncology clinics located within a regional cancer centres	Case report	Head and neck	Any	FACE-Q Head and Neck Cancer Module, Neck Dissection Impairment Index, Skindex-16, Skull Based Inventory, and pain scale	Every outpatient visit (duration not specified)	NR	Summary reports
Sundberg 2017 [43]	Interaktor	Sweden, university hospitals	Non-randomized, historically controlled study	Prostate	Active treatment and follow-up	Symptoms based on the memorial symptom assessment scale including, bladder and bowel function, fatigue, pain, anxiety, distress, sleep, and flushing	Daily for 8–11 weeks	Self-management and score history	Alerts
Taarnhoj 2020 [44]	e-Boks system and AmuFlex software	Denmark, outpatient oncology clinics located within university hospitals	Single-arm intervention	Bladder	Active treatment	EORTC QLQ-C30 and QLQ-BLM30, HADS, and CTCAE	Weekly until treatment completion	NR	Summary reports
Tolstrup 2020 [45, 71]	NR	Denmark, outpatient clinics located within a university hospital	RCT + qualitative focus groups and interviews	Melanoma	Active treatment	PRO-CTCAE	Weekly for 24 weeks	System message	Summary report
van der Hout 2020 [46]	OncoKompas	Netherlands, hospital setting	RCT	Head and neck, colorectal, breast, and lymphoma	Follow-up	EORTC	Frequency and duration not specified (patients could use the system on a voluntary basis)	Self-management	NR
van Eenbergen 2019 [47]	BijKanker	Netherlands, hospital setting	Single-arm intervention	Any	Active treatment	Nurse Problem Analysis	Weekly until treatment completion	Self-management, score history, and communication	Summary reports
Wagner 2015 [58]	Assessment Center software	USA, outpatient oncology clinic located with a cancer center	Single-arm intervention	Gynecologic	Any	PROMIS, Distress Thermometer, Problem Checklist, Patient-Generated Subjective Global Assessment	Every outpatient visit (duration not specified)	NR	Alerts, summary reports, and communication
Wu 2016 [62, 86]	Patient Viewpoint	USA, outpatient oncology clinic located with a cancer center	Single-arm intervention	Breast and prostate	Active treatment	PROMIS, EORTC QLQ C30 and QLQ-BR23, EPIC, and SCNS	Every 2 weeks (duration not specified)	NR	Summary reports and recommended actions
Zebralla 2020 [48]	OncoFunction	Germany, outpatient clinic	Single-arm intervention + qualitative interviews	Head and neck	Follow-up	Visual analogue (pain), EAT10, PHQ-9, GAD-2, EORTC QLQ C30	Every outpatient follow-up visit until discharged by oncology team	NR	Summary reports
Zylla 2020 [65]	NR	USA, urban community cancer center	Single-arm intervention	Any non-hematologic cancers	Active treatment	Patient-reported symptom monitoring tool	Every 2 weeks for 12 weeks	NR	Summary reports

*STAR* Symptom Tracking and Reporting, *PRO-CTCAE* Patient-reported outcomes-common terminology criteria for adverse events, *ECOG* Eastern Cooperative Oncology Group, *EQ-5D* EuroQol- 5 Dimension, *PACE* Patient Assessment, Care and Education, *FACT-G* Functional Assessment of Cancer Therapy – General, *FACT-B* Functional Assessment of Cancer Therapy – Breast, *MDASI* MD Anderson Symptom Inventory, *FACIT-Fatigue* Functional Assessment of Chronic Illness Therapy – Fatigue, *EORTC QLQ* European Organization for the Research and Treatment of Cancer Quality of Life Questionnaire, *CHES* Computer-based Health Evaluation System, *ESAS* Edmonton Symptom Assessment System, *ESRA-C* Electronic Self-Report Assessment– Cancer, *RCT* Randomized controlled trial, *ASyMS* Advanced Symptom Monitoring and Management System, *PROMIS* Patient-Reported Outcomes Measurement Information System, *DART* Distress assessment and response, *PHQ* Patient health questionnaire, *GAD-7* Generalized anxiety disorder assessment, *SDI* Social Difficulties Inventory, *EPIC* Expanded Prostate Cancer Index, *SCNS* Supportive care needs survey, *HADS* Hospital Anxiety and Depression Scale, *PROMPT-Care* Patient Reported Outcome Measures for Personalized Treatment and Care, *iComPAsS* Internet-based Computerized Patient Assessment System, *ORION* Outcome Registry Intervention and Operation Network, *FT12* Financial toxicity, *COST-FACIT* COmprehensive Score for financial Toxicity, *EAT10* Eating Assessment Tool, *BPI* Brief pain inventory, *iPROMOS* Integrating Patient Reported Outcomes in a Medical Oncology Setting, *eRAPID* Electronic patient self-Reporting of Adverse-events: Patient Information and aDvice, *CTAQ* Chemotherapy Toxicity Self- Assessment Questionnaire, *REMOQOL* Routine Electronic Monitoring of Health-Related Quality of Life

### ePSM intervention characteristics

Most interventions did not have a fixed (e.g., weekly or monthly) surveillance schedule for patients (*n* = 25, 54%), with most asking patients to complete ePROs at any outpatient visit [14, 15, 28, 30–34, 36, 41, 46, 48, 51–53, 56–58, 60, 61, 63, 64, 66, 69, 70]. Some interventions allowed clinicians to personalize the frequency of reporting for patients or asked patients to report based on their preference (*n* = 3, 7%) [28, 46, 70]. Of interventions with fixed surveillance schedules, reporting varied from daily (*n* = 6, 13%) [35, 38–40, 43, 59], weekly or bi-weekly (*n* = 11, 24%) [27, 37, 44, 45, 47, 49, 50, 54, 62, 65, 68], monthly (*n* = 3, 7%) [29, 55, 67], and quarterly (*n* = 1, 2%) [42]. The duration of surveillance ranged from 1 month (*n* = 2, 4%) [50, 59], greater than 1 to 6 months (*n* = 13, 28%) [15, 27, 28, 35, 38, 41, 43, 45, 49, 57, 64, 65, 70], greater than 6 to 2 years (*n* = 5, 11%) [36, 42, 54–56], and up to 5 years after completing treatment (*n* = 1, 2%) [31]. Over half of the interventions did not specify a fixed duration of surveillance (*n* = 25, 54%), but rather described that patients were followed until they completed treatment or were no longer being followed by the oncology team [14, 29, 30, 32–34, 37, 39, 40, 44, 46–48, 51–53, 58, 60–63, 66–69].

The ePSM system features specified for each study in Table 1 are further described in Additional file 5. The most common patient-targeted features included automatically providing patients with self-management material to address symptoms (*n* = 17, 37%) [15, 27, 28, 30, 35, 38–40, 43, 46, 47, 50, 54, 59, 64, 67, 68], the option to view how scores had changed over time (*n* = 10, 22%) [15, 27, 30, 35, 38, 40, 43, 47, 59, 69], and an automated message on remote systems informing them that their scores were not being monitored by their provider with appropriate contact information if further support was required (*n* = 9, 20%) [14, 27, 35, 45, 49, 50, 52, 56, 57]. Other features included the ability to message providers or administrators to ask questions or request an e-consult (*n* = 4, 9%) [42, 47, 59, 70], general education about treatments and potential side effects, and/or information about patients’ legal rights (*n* = 3, 7%) [28, 59, 66], and the ability to view their circle of care including a list of attending physicians and their contact information (*n* = 2, 4%) [40, 70].

The most common provider-targeted features included the option to view summary reports of patients’ symptoms, including graphs indicating symptom thresholds and severity (*n* = 41, 89%) [14, 15, 27, 29, 31–42, 44, 45, 47–54, 56–70], alerts for symptoms that had breached a specified threshold (*n* = 15, 33%) [14, 27, 35, 38–40, 43, 49, 50, 52, 54, 55, 57, 58, 67], the provision of recommended actions and referrals to facilitate symptom management (*n* = 5, 11%) [40, 54, 62, 63, 67], and the ability to send messages to patients, such as reminders, prescriptions, and appointment schedules (*n* = 3, 7%) [35, 58, 70].

### Implementation theories, models, and frameworks

Ten studies (22%) reported using a theory, model, or framework to guide implementation planning or evaluation. Process models were used by six studies (14%) [39, 40, 51, 53, 73, 83], such as the Medical Research Council framework for the development of complex interventions and the Knowledge-to-Action Framework [87, 88]. Models from the quality improvement literature were also utilized by two studies (5%) [15, 63]. The integrated Promoting Action on Research Implementation in Health Services [89] and the implementation outcomes taxonomy [23] were the only determinant and evaluation frameworks utilized [50, 69]. Lastly, classic theories were used by two studies (5%) [51, 70], including the Diffusion of Innovations theory [90] and the Self-Determination Theory.

### Implementation strategies

A total of 26 different implementation strategies were described within the included studies. Of these, there were a total of 153 reports of their use across the 46 interventions. The median number of discrete implementation strategies reported within interventions was 3 (interquartile range 2–4). The implementation strategies used among the included interventions are displayed in Additional file 6. Of the 153 reports of use, the strategies used most frequently were those within the cluster of train and educate stakeholders (*n* = 55, 36%) [14, 15, 27–31, 33–35, 39–45, 47, 49–51, 54, 56, 57, 60–64, 68–70, 72], followed by change infrastructure (*n* = 28, 18%) [27, 29, 31, 32, 36, 44, 48, 51–53, 55–58, 60–65, 67–70], engage consumers (*n* = 24, 16%) [15, 27, 30, 31, 37–39, 43, 49, 50, 53–58, 60, 62, 64–68], develop stakeholder interrelationships (*n* = 21, 14%) [27, 30, 34, 38–40, 50, 51, 55, 60, 63, 68, 69], use evaluative and iterative strategies (*n* = 12, 8%) [29, 30, 40, 51, 60, 63, 69], provide interactive assistance (*n* = 8, 5%) [28, 30, 34, 43, 44, 46, 55, 69], support clinicians (*n* = 3, 2%) [31, 50], and utilize financial strategies (*n* = 1, 1%) [30].

Among the 46 ePSM interventions, the most common discrete implementation strategies utilized included conduct educational meetings (*n* = 25, 54%) [14, 15, 28–31, 35, 39, 40, 42, 43, 45, 47, 49–51, 54, 56, 57, 60–62, 68, 69, 72], distribute educational materials (*n* = 20, 43%) [14, 28, 30, 33–35, 40, 41, 44, 47, 50, 51, 57, 60–62, 64, 68, 69, 72], change record systems (*n* = 19, 41%) [27, 29, 31, 32, 36, 44, 51–53, 55, 58, 60–62, 65, 67–70], intervene with patients to enhance adherence and uptake (*n* = 19, 41%) [15, 31, 37, 43, 49, 53–58, 60, 62, 64–68], change physical structure and equipment (*n* = 9, 20%) [31, 32, 48, 56, 57, 60, 61, 63, 64], and provide local technical assistance (*n* = 8, 17%) [28, 30, 34, 43, 44, 46, 55, 69].

### Implementation outcomes

The median number of implementation outcomes measured per study was 3, ranging from 1 to 6. The most frequently reported outcomes were feasibility (*n* = 33, 72%) [14, 15, 27–29, 31–34, 37–42, 44, 45, 48, 49, 52–57, 61–63, 65–69] and acceptability (*n* = 31, 67%) [29, 32–39, 41, 45, 47–51, 54–57, 59–69], followed by appropriateness (*n* = 18, 39%) [31, 34–36, 38, 39, 45, 47, 50, 51, 54, 55, 60, 61, 63, 66–68], fidelity (*n* = 18, 39%) [29, 30, 35, 38, 40, 44, 46, 48, 49, 55, 56, 58, 60, 61, 65, 67, 69, 70], and penetration (*n* = 16, 35%) [15, 32, 33, 35, 41, 46, 48, 51–53, 56, 57, 60, 63, 69, 70]. Very few studies reported on cost (*n* = 4, 9%) [35, 40, 46], adoption (*n* = 2, 4%) [30, 63], or sustainability (*n* = 1, 2%) [63]. Studies used various approaches to measure implementation outcomes, including the use of surveys (*n* = 26, 57%) [27, 29–32, 34, 35, 39–41, 45, 47–49, 51, 54–57, 59, 60, 62–64, 68, 69], ePSM system data and analytics (*n* = 23, 50%) [15, 28, 29, 32–35, 37, 38, 40, 41, 44, 46, 49, 52, 56, 61–63, 65, 67, 68, 70], qualitative interviews or focus groups (*n* = 19, 41%) [27, 29, 31, 32, 35, 36, 38, 39, 42, 45, 50, 55, 61–63, 65, 66, 68, 69], administrative data (*n* = 5, 11%) [27, 32, 35, 41, 63], and field notes and observations (*n* = 3, 7%) [32, 34, 69].

### Implementation barriers and facilitators

Operationalized definitions for each CFIR domain and construct, synthesized descriptions for the barriers and facilitators identified, and the proportion of studies coded within each construct are outlined in Table 2. The most commonly reported domains were intervention characteristics (*n* = 29, 63%) [27, 29–34, 36, 38, 39, 41, 45, 47, 49–51, 54–57, 60–66, 68, 69], inner setting (*n* = 22, 48%) [29–32, 34, 36, 38, 42, 45, 50–52, 55, 56, 60–63, 65, 66, 68, 69], and outer setting (*n* = 19, 41%) [29–32, 34, 36, 38, 39, 45, 54, 59, 61–66, 68, 69]. The characteristics of individuals (*n* = 16, 35%) [29–32, 34, 36, 38, 39, 45, 54, 59, 61–66, 68, 69] and process (*n* = 14, 30%) [30–32, 34, 36, 38, 39, 50, 51, 61–63, 68, 69] were less frequently reported. A total of 17 of the 39 CFIR constructs were identified across the 46 interventions. The barriers and facilitators in the context of the five CFIR domains and the most relevant constructs are presented below.

**Table 2:** Determinants to implementation reported by the included interventions

CFIR domain and construct^a^	Barriers	Facilitators	*n* (%)
**1. Intervention Characteristics** *Attributes of the ePSM that is being implemented*			29 (63)
**Complexity** *Ease/difficulty of using the system (patients and staff)*	• Too many questions to complete • Difficulties understanding the questions • High volume of symptom alerts • ePRO reporting is too frequent • Difficulties interpreting ePRO scores • Challenges navigating the system and locating ePRO scores and/or graphical results	• Acceptable and appropriate length and duration of ePROs • Acceptable frequency of ePRO reporting • Questions are clear and easy to understand • User-friendly system that is easy to navigate	25 (54)
**Relative Advantage** *Benefits of using the ePSM versus existing assessment practices*	• ePROs are redundant with questions asked during clinic visit • ePSM information provided to patient conflicts or is redundant with information provided during the clinic visits	• Perceived improved symptom identification and management • Perceived improved patient-provider communication • Ability to personalize discussions with patients	23 (50)
**Design Quality & Packaging** *Visualization and presentation of symptom scores, services provided/recommended, and overall design of the system*	• Inability to view history of ePRO scores • Lack of clarity for whether high scores or specific colours displayed indicate better or worse status • Self-management material related to symptoms of concern are not highlighted or specified	• Clear and appealing visualization and presentation of past ePRO scores in graphical format • Ability to view a list of resources recommended to patients	14 (30)
**Adaptability** *The ability/need to have an ePSM that can be adapted and personalized*	• Inability to modify and tailor the questions, timing of reporting, and content provided to patients	• Ability to tailor the questions provided to patients based on their responses	5 (11)
**Cost** *Costs associated with ePSM implementation*	• Extra costs needed to implement the ePSM are not covered by health insurers or government	NR	2 (4)
**2. Inner Setting** *The organizational and cultural contexts in which ePSMs are implemented*			22 (48)
**Implementation Climate** *Absorptive capacity for change, shared receptivity to ePSM implementation, and the extent to which ePSM will be supported and compatible within existing processes in the organization*	• ePSM is not integrated with electronic medical record and staff need to log into a separate system to access ePRO scores • Concerns ePSM implementation will increase workloads • Concerns ePSM implementation will prolong visit times • Previous unsuccessful experiences with implementation initiatives • Limited to no experience using ePROs	• ePSM is integrated with electronic medical record and ePROs are easily accessible to staff • Workloads are not increased • Clearly communicated and ongoing monitoring of implementation goals	20 (43)
**Readiness for Implementation** *Organizational commitment to ePSM implementation including leadership engagement, available resources, and access to appropriate and sufficient information about the ePSM*	• Lack of time for staff to use ePROs during clinic visits • Lack of information about the ePSM for clinic staff to use during clinic visits (guidelines and algorithms on how to manage high scores) • Insufficient resources respond to symptoms identified by ePSM	• Support from leadership and senior management • Availability of volunteers to educate and support patient completing ePROs in clinic	12 (26)
**Networks & Communications** *Nature and quality of social networks and communication within an organization*	• Limited communication between disciplines • Concerns other disciplines may not engage in the implementation effort and lead to a disproportionate burden on some staff	• ePSM implementation increased teamwork among clinical staff • Agreement about the division of roles and responsibilities among implementation, clinical, and software teams	3 (7)
**3. Outer Setting** *The broader context within which an organization implementing an ePSM is situated (i.e., external influences)*			19 (41)
**Patient Needs & Resources** *Specific needs of patients that were/were not met or that demonstrate a need for an ePSM*	• Perceived lack of relevance and usefulness of the ePSM questions and/or content • ePRO and/or self-management material are not tailored to the individual patient	• ePSM questions and content is perceived as relevant, useful, and meaningful for patients • Perceived improved attention and insight into patient symptoms • ePSM use provides a sense of reassurance and reduced uncertainty about symptoms • ePSM use provides a sense of empowerment and control	18 (39)
**Cosmopolitanism** *Degree to which an organization implementing ePSM is networked with other external organizations*	• Limited connections with other services successfully using ePROs	• Ability to involve other providers such as patients’ general practitioners to ensure that key information regarding their clinical care was not overlooked	2 (4)
**4. Individual Characteristics** *Attributes of the individuals involved in the implementation of an ePSM*			16 (35)
**Knowledge & Beliefs** *Attitudes toward and value placed on the ePSM, including familiarity with principles related to the ePSM*	• Lack of knowledge and awareness about the ePSM features, content, and aims • Lack of knowledge on how to complete ePROs • ePROs not perceived as valuable for clinical practice	• Knowledge and understanding of ePSM features, content, and aims • Strong professional values for the use of ePROs for clinical practice • Belief that symptom management is within staff’s scope of responsibilities	8 (17)
**Other Personal Attributes**	• Lack of comfort, experience and access to technology and internet • Patient is too ill to report symptoms	• Prior experience with using technologies and internet	10 (22)
**5. Process** *Active change processes used to implement ePSMs*			14 (30)
**Engaging** *Attracting and involving appropriate individuals in ePSM implementation, including opinion leaders, champions, change agents, key stakeholders, and patients*	• Educational strategies (e.g., handouts) not used by patients • Information provided to patients was not clear • Insufficient amount of training provided to patients and staff • Inability for patients to self-register to the ePSM	• Engaging a broad group of stakeholders • Involvement of champions and respected peers • Acceptable duration and timing of educational and training strategies • Provision of reminders for patients and/or staff to use ePSM • Availability of support for practical and technical issues	13 (28)
**Reflecting & Evaluating** *Feedback about the process and quality of ePSM implementation*	• Implementing the ePSM across many different sites at once • Clinic staff are not provided with a specific strategy to incorporate ePROs into workflow	• Use of a flexible and iterative approach to implementation • Use of data and regular meetings with stakeholders to track and monitor implementation	4 (9)

^a^Includes constructs reported by at least two studies. *ePRO* Electronic patient-reported outcome, *ePSM* Electronic prospective surveillance model, *NR* Not reported, *n* (%) Signifies the frequency and percentages of the 46 ePSM interventions included in the review

#### Intervention characteristics

The most common constructs for intervention characteristics (i.e., key attributes of the ePSM that is being implemented) were complexity and relative advantage. Within complexity, barriers centered on the complexity of the surveillance system design. From a provider perspective, this included a high volume of patient responses or alerts provided about patients’ symptoms [31, 32, 45, 47, 50, 54, 61, 66, 68] and interpreting symptom scores [31, 34, 41, 62]. From a patient perspective, complex systems presented challenges in understanding what was being asked of them [31, 34, 41, 62]. Difficulty in navigation of the system was a barrier for both patients and providers [32–34, 36, 39, 61, 62, 68]. Alternatively, facilitators included perceptions that the duration and frequency of completing the ePROs were appropriate [31, 34, 45, 50, 54, 57, 60, 65], the ability to understand the questions asked [34, 49, 54, 64], and perceptions that the system was easy to use [27, 33, 38, 39, 45, 47, 49, 54, 56, 57, 62, 64, 65].

For relative advantage, barriers included perceptions that the ePROs and/or the self-management material were redundant and/or conflicting with assessments and information provided by the oncology team during clinic visits [39, 50, 51, 55, 61, 62, 68]. Facilitators included perceptions that the ePSM improved symptom identification and management [29, 32, 33, 38, 39, 45, 47, 51, 57, 61, 62, 65, 66], improved communication and quality of discussions between patients and providers [29, 33, 45, 51, 54, 56, 57, 60, 63–66], and allowed the provider to personalize the clinic visit based on the ePRO scores [45, 61, 65, 66, 68].

#### Inner setting

The most common determinants within the inner setting (i.e., the specific organizational and cultural contexts in which ePSMs are implemented) were implementation climate, and readiness for implementation. The implementation climate is most often related to the compatibility between the ePSM and existing workflows. Barriers included not integrating the ePSM with the electronic medical record, as clinic staff had to log into a different system to view patients’ ePRO results [29, 45, 50, 62]. Additionally, barriers included perceptions that implementing an ePSM would result in an increased workload due to having to review ePRO results before a clinic visit, potential challenges integrating the management of symptom alerts into existing communication channels, and the potential to prolong visit times [31, 36, 38, 42, 50, 51, 61, 63, 66, 68, 69]. Alternatively, facilitators included integrating the ePSM with the electronic medical record [31, 32, 45, 52, 61, 62], and perceptions that workloads among clinic staff were not increased as a result of implementing an ePSM [42, 65].

Barriers related to the readiness for implementation involved a lack of resources to implement the ePSM. This included reports of insufficient time for clinicians to use ePRO scores during clinic visits [32, 36, 56, 62, 69], and concerns that the center would not have the necessary resources to respond to symptoms identified by the ePSM [31, 63, 69]. Studies reported a lack of information related to the ePSM to facilitate its use, such as explanations about ePRO scores and guidance for assessing and managing high scores [29, 62, 63]. Facilitators included having clear, supportive, and committed leadership from senior staff and managers [36, 51, 60, 61, 69], as well as the availability and involvement of volunteers to provide education and support to patients completing ePROs in-clinic [63].

#### Outer setting

Barriers and facilitators for the outer setting (i.e., the broader context within which an organization implementing an ePSM is situated) were almost exclusively related to the extent to which patients’ needs were met by the setting that implemented the ePSM (i.e., patient needs and resources). Barriers included perceptions of the lack of usefulness of the ePROs and self-management material [32, 36, 45, 66, 68], particularly when patients’ responses to the ePROs were not mentioned during their clinic visit [36, 61, 62], as well as perceptions that the ePROs and self-management material were not sufficiently tailored to the individual patient [30, 68]. Facilitators included perceptions that the ePROs were relevant and meaningful for patients [29, 34, 38, 45, 54, 59, 62, 64–66], as well as perceptions that using the system gave patients a sense of reassurance about their wellbeing and provided them with a sense of empowerment and control [31, 38, 39, 54, 62, 68]. Additionally, facilitators included beliefs that using the ePSM provided patients and providers with greater attention and insight into their symptoms, including the ability for patients to remember their symptoms between clinic visits and the ability for staff to provide appropriate referrals [29, 31, 32, 45, 63–66, 68].

#### Characteristics of individuals

The most common determinants were knowledge and beliefs, and personal attributes. Barriers identified for knowledge and beliefs included a lack of knowledge among patients and clinic staff about the ePSM features and how to complete the ePROs [29, 36, 50, 69], as well as beliefs that the use of ePROs was not valuable [34, 61]. Facilitators identified included an understanding of the content and features of the ePSM [50, 56], as well as when and how to complete the ePROs [50]. Additionally, facilitators included strong professional values for using ePROs for clinical practice and beliefs that symptom management is within a provider’s scope of responsibilities [61, 63, 69].

For other personal attributes of patients, barriers included a lack of comfort and experience with technology [30, 41, 47, 54, 72], limited access to reliable internet or electronic devices [48, 55], and feeling too ill to report symptoms [29, 57, 62]. Alternatively, facilitators included prior experience of patients using connected technologies, thus more likely to demonstrate greater usability and use of the system [48, 54, 57].

#### Process

The most common determinants for the process domain (i.e., the stages and active change processes used to implement ePSMs) included engaging stakeholders and patients. Barriers included perceptions that the educational strategies such as handouts were not used by patients or that information provided was not clear [39, 50, 62], perceptions that patients did not receive sufficient training and were unaware of various features of the system [39, 50], and that patients had to register through their health care provider rather than being able to self-register to the system [30]. Facilitators included engaging a broad group of stakeholders, including the involvement of respected peers [34, 51, 63, 69], perceptions that the duration and timing of the education and training for patients or staff were appropriate [34, 39, 50], and beliefs that the ePSM was clearly explained to stakeholders [32, 61, 62]. Facilitators included building patient and clinician capacity and confidence to use the system through quality education and training strategies and the availability of support to resolve practical and technical issues [34, 50, 51, 69]. Lastly, facilitators included the use of reminders for patients and clinicians to use the system [32, 38, 62].

## Discussion

This scoping review synthesized 46 ePSMs to summarize the approach to implementing this intervention in routine cancer care. The findings provide a foundation for informing and improving the implementation of ePSMs, including selecting implementation strategies, planning for barriers and facilitators, and evaluating key implementation outcomes.

The use of TMFs has been strongly advocated for in implementation science to guide the planning, process, and evaluation of moving evidence-based practices into action. However, a minority of included studies reported using any. This may be partly because many included studies did not identify as implementation science studies and were rather descriptions of implementation in practice. Implementation science is a relatively young field, and we anticipate the use of these may increase in the future. Their use in future implementation efforts may provide a better understanding of the steps taken during implementation and how or why implementation was or was not successful.

Feasibility and acceptability were commonly reported implementation outcomes, while adoption, cost, and sustainability were seldom reported. Implementing an intervention involves various steps, and certain outcomes may be prioritized during different phases of implementation [99]. Capturing outcomes such as feasibility and acceptability are recommended before or during the initial implementation of an intervention [23]. The lack of reported use of adoption, cost, and sustainability can be explained in part because research on the implementation of ePSMs is still in its infancy; however, these outcomes should be a major focus in reporting future implementation efforts. Many articles in this review reported on implementing an ePSM in a single setting, rather than investigating the scale or spread across oncology clinics (i.e., adoption). While sustainability can be assessed during the early stages of implementation to identify areas that require improvement [100], research typically focuses on the early stages of implementation and little attention is paid to sustaining interventions [91]. This can also explain the lack of implementation strategies identified that were focused on the sustainability of ePSMs.

Recently published clinical practice guidelines on the role of patient-reported outcome measures (PROMs) in oncology highlight the need for improved evidence regarding optimal implementation strategies [92]. Our findings provide a list of implementation strategies used for ePSMs, their frequency of use, action targets, and when they were used in the implementation process. The most frequently used categories of implementation strategies were educating stakeholders, changing infrastructure, and engaging patients. Interestingly, there is only moderate alignment between the most used strategies and reported determinants of implementation in the included studies. Within the field of implementation science, it is recommended to use an assessment of barriers and facilitators to identify relevant implementation strategies; future studies should carefully consider local contextual determinants of implementation before embarking on an implementation project.

The most frequently reported determinant domains were the intervention characteristics, outer and inner settings. At the level of the intervention, implementors should consider the complexity of the system by ensuring patients and providers consider it clear, easy to use, and perceive the duration and frequency of reporting to be acceptable. This could be achieved using strategies within the cluster of engaging consumers, and adapting and tailoring to context; however, these were not among the most commonly reported strategies in this review. Likewise, when designing the system, it is essential to ensure repeated ePROs are displayed longitudinally using clear graphical depictions of the patient’s status over time. To address identified barriers to ePSM implementation, particular emphasis should be placed on highlighting the relative advantages of using the ePSM compared to existing clinical practices (e.g., improved symptom management through early identification and communication).

Key factors influencing implementation from the inner setting domain were related to the implementation climate and readiness for implementation; however, evaluative and iterative strategies such as conducting a local needs assessment and assessing for readiness were seldom reported in the included studies, reflecting an area of opportunity for future work. A recent systematic review emphasizes the importance of assessing the implementation climate, demonstrating that features such as organizational culture, leadership, and resources influence the implementation of interventions in healthcare settings [93]. Factors such as management support, organizational priorities, and organizational buy-in have been identified as key factors for sustaining cancer survivorship interventions [94, 95]. Therefore, implementers should consider how the implementation of an ePSM can be integrated within existing workflows and other electronic systems used in the setting and obtain support from senior leadership.

Meeting the needs of patients was another critical determinant of successful implementation. Implementors should consider whether patients find the screening questions and information relevant to their cancer care and the types and levels of resources available to patients that may require support identified by the ePSM. While sites may have concerns about a lack of dedicated programs and cancer rehabilitation clinicians to meet the needs of patients [7], as was reported in several studies in this review, directing patients to self-management resources and eHealth interventions may address many accessibility barriers to meet the needs of patients [95–97]. Furthermore, since many of the facilitators identified rely on a contextual understanding of patient/provider needs, preferences, and existing workflows, engaging a broad group of stakeholders throughout implementation using a flexible and iterative approach is likely key to successful ePSM implementation. Strategies in the clusters of adapting and tailoring to context, developing stakeholder interrelationships, and changing infrastructure will be important to address these determinants. Within included studies, the role of healthcare coverage and health system arrangement (as part of the outer setting) was not identified as a determinant of implementation, likely because nearly all studies were implemented within a single system. To scale and spread these interventions to other jurisdictions, one would expect these outer setting factors to be of great importance. Cost was reported as a barrier in two studies conducted in Europe; however, only four studies collected and reported on cost as an outcome. Future studies examining the implementation of an ePSM should consider capturing perspectives from patients, providers, and administrators on costs, policies, and regulatory environments that may hinder or enable implementation.

Powell et al. [22] identified implementation strategies in the “Go-Zone” quadrants—those rated as most important for implementation and the most feasible were placed into quadrant 1. Interestingly, most of these strategies fell within the cluster of use evaluative and iterative strategies, with the most highly rated for both importance and feasibility being “assess readiness and identify barriers and facilitators,” “audit and feedback,” and “purposefully re-examine implementation.” Within this review, the most often reported strategies were within the “train and educate stakeholders” cluster, although knowledge was not identified as a critical barrier, nor was education or training an important facilitator. While some strategies, such as conduct ongoing training and providing ongoing consultation, were rated as highly important by the expert group, others such as developing and distributing educational materials and conducting educational meetings and outreach visits, while highly rated for feasibility, were ranked lower in importance. It is important to note that these rankings were based on expert opinion, and to date, there is scant literature to objectively determine which implementation strategies are “best”.

Previous reviews have identified similar facilitators to implementation such as patients’ acceptability to report symptoms, the ability for PROMs to enable earlier detection of symptoms, and improving patient-provider communication [16]. Additionally, previous reviews have identified similar barriers such as patient and clinician time, knowledge to interpret and act on scores, and challenges integrating PROMs into workflows [16, 97]. However, two critical factors differentiate previous reviews from our scoping review. First, our study adds to the literature on determinants of routine use of PROMs by using a well-known implementation science framework (i.e., CFIR) to categorize barriers and facilitators. This provides a comprehensive understanding of implementation determinants and may facilitate selecting strategies to address these domains and constructs. Furthermore, our review was solely focused on electronic reporting of symptoms and included additional findings related to implementation, such as the strategies, outcomes, and TMFs used.

Given the novelty of implementation research for ePSMs, this review may not have captured every potential strategy or determinant to implementation. As many of the included studies did not identify as implementation science studies, it is likely that other implementation strategies may have been used but not reported and that the focus on acceptability and feasibility implementation outcomes, as opposed to adoption, cost, and sustainability may be explained in part by the inclusion of these studies. Additionally, wide variation was found with respect to the characteristics of the ePSMs, including the ePROs used, patient populations examined, and treatment phases under study. It is possible that different determinants and, thus, the most relevant associated strategies may vary greatly by the characteristics of the ePSM. For example, most ePSMs included in this review were designed exclusively for patients on active treatment who were receiving chemotherapy, and few studies examined use in the palliative care setting. Future research may provide insight into similarities and differences in implementation across patient populations and settings and provide recommendations for adapting the implementation of ePSMs to meet unique needs.

## Conclusions

This scoping review provides a foundation for future planning and evaluation of the implementation of ePSMs in oncology. These findings can facilitate the selection of implementation strategies; however, future studies should consider testing the effectiveness of these strategies. Advancing this knowledge through high-quality implementation science research will provide robust evidence on the effects of various strategies and their mechanism of action for successful implementation [98]. The findings highlight the need to consider the use of implementation science TMFs and provide insight into implementation determinants that researchers and implementors should consider.

## Supplementary Information

**Additional file 1.** Preferred Reporting Items for Systematic reviews and Meta-Analyses extension for Scoping Reviews Checklist.

**Additional file 2.** Search Strategies.

**Additional file 3.** Adapted ERIC descriptions for implementation strategies used in the included interventions.

**Additional file 4.** Adapted descriptions for implementation outcomes reported by the included interventions.

**Additional file 5.** Description of electronic prospective surveillance model system features.

**Additional file 6.** Clusters and discrete implementation strategies used in the included interventions.

## Data Availability

The dataset used and analyzed during the current study are available from the corresponding author on reasonable request.

## Abbreviations

PSM: Prospective Surveillance Model
ePSM: Electronic Prospective Surveillance Model
ePROs: Electronic patient-reported outcomes
TMF: Theory, model, or framework
ERIC: Expert Recommendations for Implementing Change
CFIR: Consolidated Framework for Implementation Research
PROMs: Patient-reported outcome measures
