# Supplementary material for: Implementation of electronic prospective surveillance models in cancer care: a scoping review
Source: Implement Sci. 2023 Apr 26;18:11. doi: 10.1186/s13012-023-01265-4 (PMC10134630; doi:10.1186/s13012-023-01265-4)
Supplement: Supplementary file 2 — Additional file 2. Search Strategies. [file 13012_2023_1265_MOESM2_ESM.docx]

**Additional File 2. Search Strategy**

**Ovid MEDLINE(R) ALL 1946 to February 01, 2021**

| **#** | Searches | **Results** | **Type** |  |  |  |
| --- | --- | --- | --- | --- | --- | --- |
|  | | | | | | |
| 1 | exp Neoplasms/ | 3410563 | Advanced |  |  |  |
| 2 | Cancer Survivors/ | 4543 | Advanced |  |  |  |
| 3 | exp Oncologists/ | 1102 | Advanced |  |  |  |
| 4 | exp Medical Oncology/ | 23957 | Advanced |  |  |  |
| 5 | Surgical Oncology/ | 458 | Advanced |  |  |  |
| 6 | Radiation Oncology/ | 4263 | Advanced |  |  |  |
| 7 | Psycho-Oncology/ | 123 | Advanced |  |  |  |
| 8 | Oncology Nursing/ | 7989 | Advanced |  |  |  |
| 9 | Oncology Service, Hospital/ | 1484 | Advanced |  |  |  |
| 10 | Cancer Care Facilities/ | 5522 | Advanced |  |  |  |
| 11 | neoplas*.tw,kf. | 410198 | Advanced |  |  |  |
| 12 | paraneoplas*.tw,kf. | 11207 | Advanced |  |  |  |
| 13 | cancer*.tw,kf. | 1892522 | Advanced |  |  |  |
| 14 | tumo?r*.tw,kf. | 1775193 | Advanced |  |  |  |
| 15 | onco*.tw,kf. | 341375 | Advanced |  |  |  |
| 16 | metast*.tw,kf. | 535099 | Advanced |  |  |  |
| 17 | malignan*.tw,kf. | 597297 | Advanced |  |  |  |
| 18 | aberrant crypt foci.tw,kf. | 1536 | Advanced |  |  |  |
| 19 | acanthoma*.tw,kf. | 696 | Advanced |  |  |  |
| 20 | acrospiroma*.tw,kf. | 79 | Advanced |  |  |  |
| 21 | adamantinom*.tw,kf. | 1108 | Advanced |  |  |  |
| 22 | adenocarc*.tw,kf. | 155454 | Advanced |  |  |  |
| 23 | adenofibrom*.tw,kf. | 561 | Advanced |  |  |  |
| 24 | adenolymphom*.tw,kf. | 462 | Advanced |  |  |  |
| 25 | adenomat*.tw,kf. | 19335 | Advanced |  |  |  |
| 26 | adenomyo*.tw,kf. | 4166 | Advanced |  |  |  |
| 27 | adenosarcom*.tw,kf. | 590 | Advanced |  |  |  |
| 28 | adenosquam*.tw,kf. | 2896 | Advanced |  |  |  |
| 29 | ameloblastom*.tw,kf. | 3546 | Advanced |  |  |  |
| 30 | androblastom*.tw,kf. | 99 | Advanced |  |  |  |
| 31 | angiofibrom*.tw,kf. | 2211 | Advanced |  |  |  |
| 32 | angiokeratom*.tw,kf. | 1041 | Advanced |  |  |  |
| 33 | angiolipom*.tw,kf. | 627 | Advanced |  |  |  |
| 34 | angioma*.tw,kf. | 11532 | Advanced |  |  |  |
| 35 | angiomyolipom*.tw,kf. | 4127 | Advanced |  |  |  |
| 36 | angiomyom*.tw,kf. | 162 | Advanced |  |  |  |
| 37 | angiosarcom*.tw,kf. | 6613 | Advanced |  |  |  |
| 38 | apudoma*.tw,kf. | 301 | Advanced |  |  |  |
| 39 | arrhenoblastom*.tw,kf. | 354 | Advanced |  |  |  |
| 40 | astrocytom*.tw,kf. | 16576 | Advanced |  |  |  |
| 41 | blastom*.tw,kf. | 9777 | Advanced |  |  |  |
| 42 | Bowen*.tw,kf. | 3187 | Advanced |  |  |  |
| 43 | Brenner*.tw,kf. | 1538 | Advanced |  |  |  |
| 44 | Buschke-Lowenstein*.tw,kf. | 264 | Advanced |  |  |  |
| 45 | carcin*.tw,kf. | 836689 | Advanced |  |  |  |
| 46 | cementoma*.tw,kf. | 212 | Advanced |  |  |  |
| 47 | chemodectomas*.tw,kf. | 303 | Advanced |  |  |  |
| 48 | cholangiocarcin*.tw,kf. | 14183 | Advanced |  |  |  |
| 49 | chondroblastom*.tw,kf. | 1074 | Advanced |  |  |  |
| 50 | chondroma*.tw,kf. | 4428 | Advanced |  |  |  |
| 51 | chordoma*.tw,kf. | 4176 | Advanced |  |  |  |
| 52 | chondrosarcom*.tw,kf. | 8312 | Advanced |  |  |  |
| 53 | choriocarcin*.tw,kf. | 7169 | Advanced |  |  |  |
| 54 | craniopharyngioma*.tw,kf. | 4515 | Advanced |  |  |  |
| 55 | cystadenofibrom*.tw,kf. | 205 | Advanced |  |  |  |
| 56 | cystosarcom*.tw,kf. | 641 | Advanced |  |  |  |
| 57 | cytoma*.tw,kf. | 368 | Advanced |  |  |  |
| 58 | dermatofibrosarcom*.tw,kf. | 2020 | Advanced |  |  |  |
| 59 | desmoplas*.tw,kf. | 4989 | Advanced |  |  |  |
| 60 | dysgerminoma*.tw,kf. | 1410 | Advanced |  |  |  |
| 61 | DCIS.tw,kf. | 5229 | Advanced |  |  |  |
| 62 | DSRCT.tw,kf. | 357 | Advanced |  |  |  |
| 63 | ependymom*.tw,kf. | 5501 | Advanced |  |  |  |
| 64 | Ewing*.tw,kf. | 10068 | Advanced |  |  |  |
| 65 | fibroadenom*.tw,kf. | 3944 | Advanced |  |  |  |
| 66 | fibroepithelial*.tw,kf. | 1113 | Advanced |  |  |  |
| 67 | fibroma*.tw,kf. | 13279 | Advanced |  |  |  |
| 68 | fibrosarcom*.tw,kf. | 11957 | Advanced |  |  |  |
| 69 | FAMMM.tw,kf. | 82 | Advanced |  |  |  |
| 70 | gangliogliom*.tw,kf. | 1453 | Advanced |  |  |  |
| 71 | ganglioneurom*.tw,kf. | 2240 | Advanced |  |  |  |
| 72 | gastrinoma*.tw,kf. | 1727 | Advanced |  |  |  |
| 73 | germinoma*.tw,kf. | 1924 | Advanced |  |  |  |
| 74 | glioblastom*.tw,kf. | 40262 | Advanced |  |  |  |
| 75 | glioma*.tw,kf. | 60626 | Advanced |  |  |  |
| 76 | gliosarcom*.tw,kf. | 1169 | Advanced |  |  |  |
| 77 | glomus jugulare*.tw,kf. | 861 | Advanced |  |  |  |
| 78 | glomus tympanicum*.tw,kf. | 196 | Advanced |  |  |  |
| 79 | glucagonoma*.tw,kf. | 968 | Advanced |  |  |  |
| 80 | gonadoblastom*.tw,kf. | 831 | Advanced |  |  |  |
| 81 | GCTOB.tw,kf. | 10 | Advanced |  |  |  |
| 82 | GIST?.tw,kf. | 8277 | Advanced |  |  |  |
| 83 | hemangioendotheliom*.tw,kf. | 3081 | Advanced |  |  |  |
| 84 | hemangiom*.tw,kf. | 20572 | Advanced |  |  |  |
| 85 | hemangiopericytom*.tw,kf. | 2975 | Advanced |  |  |  |
| 86 | hemangiosarcom*.tw,kf. | 1124 | Advanced |  |  |  |
| 87 | hamartoblastom*.tw,kf. | 49 | Advanced |  |  |  |
| 88 | hepatoblastom*.tw,kf. | 3644 | Advanced |  |  |  |
| 89 | hepatoma*.tw,kf. | 29246 | Advanced |  |  |  |
| 90 | histiocytom*.tw,kf. | 5754 | Advanced |  |  |  |
| 91 | hodgkin*.tw,kf. | 69056 | Advanced |  |  |  |
| 92 | nonhodgkin*.tw,kf. | 133 | Advanced |  |  |  |
| 93 | (hutchinson* adj2 freckle*).tw,kf. | 58 | Advanced |  |  |  |
| 94 | HNPCC.tw,kf. | 2274 | Advanced |  |  |  |
| 95 | immunocytom*.tw,kf. | 608 | Advanced |  |  |  |
| 96 | incidentaloma?.tw,kf. | 2320 | Advanced |  |  |  |
| 97 | insulinoma*.tw,kf. | 6650 | Advanced |  |  |  |
| 98 | kasabach merrit*.tw,kf. | 761 | Advanced |  |  |  |
| 99 | leiomyoblastom*.tw,kf. | 400 | Advanced |  |  |  |
| 100 | leiomyom*.tw,kf. | 15615 | Advanced |  |  |  |
| 101 | leiomyosarcom*.tw,kf. | 10635 | Advanced |  |  |  |
| 102 | leukem*.tw,kf. | 242667 | Advanced |  |  |  |
| 103 | preleukem*.tw,kf. | 1505 | Advanced |  |  |  |
| 104 | leukoplak*.tw,kf. | 4876 | Advanced |  |  |  |
| 105 | li-fraumeni*.tw,kf. | 1347 | Advanced |  |  |  |
| 106 | lipoblastom*.tw,kf. | 521 | Advanced |  |  |  |
| 107 | lipoma*.tw,kf. | 15274 | Advanced |  |  |  |
| 108 | liposarcom*.tw,kf. | 6787 | Advanced |  |  |  |
| 109 | luteoma*.tw,kf. | 228 | Advanced |  |  |  |
| 110 | lymphangio*.tw,kf. | 13645 | Advanced |  |  |  |
| 111 | lymphoblastom*.tw,kf. | 369 | Advanced |  |  |  |
| 112 | lymphocytom*.tw,kf. | 347 | Advanced |  |  |  |
| 113 | lymphoma*.tw,kf. | 185692 | Advanced |  |  |  |
| 114 | lymphosarcom*.tw,kf. | 5205 | Advanced |  |  |  |
| 115 | lynch*.tw,kf. | 4627 | Advanced |  |  |  |
| 116 | macroglobulinem*.tw,kf. | 3859 | Advanced |  |  |  |
| 117 | m?croprolactinom*.tw,kf. | 862 | Advanced |  |  |  |
| 118 | mastocytom*.tw,kf. | 1865 | Advanced |  |  |  |
| 119 | mastocytos?s*.tw,kf. | 3868 | Advanced |  |  |  |
| 120 | medulloblastom*.tw,kf. | 8622 | Advanced |  |  |  |
| 121 | meigs*.tw,kf. | 873 | Advanced |  |  |  |
| 122 | melanoameloblastom*.tw,kf. | 10 | Advanced |  |  |  |
| 123 | melanoblastom*.tw,kf. | 476 | Advanced |  |  |  |
| 124 | melanocarcin*.tw,kf. | 103 | Advanced |  |  |  |
| 125 | melanoma*.tw,kf. | 122880 | Advanced |  |  |  |
| 126 | melanosis.tw,kf. | 2476 | Advanced |  |  |  |
| 127 | melanotic*.tw,kf. | 2601 | Advanced |  |  |  |
| 128 | meningiom*.tw,kf. | 21720 | Advanced |  |  |  |
| 129 | mesenchymom*.tw,kf. | 860 | Advanced |  |  |  |
| 130 | mesoblast*.tw,kf. | 740 | Advanced |  |  |  |
| 131 | mesonephrom*.tw,kf. | 123 | Advanced |  |  |  |
| 132 | mesotheliom*.tw,kf. | 16896 | Advanced |  |  |  |
| 133 | metaplas*.tw,kf. | 23559 | Advanced |  |  |  |
| 134 | micrometast*.tw,kf. | 6796 | Advanced |  |  |  |
| 135 | muir-torre*.tw,kf. | 477 | Advanced |  |  |  |
| 136 | myelolipom*.tw,kf. | 1129 | Advanced |  |  |  |
| 137 | myoepitheliom*.tw,kf. | 762 | Advanced |  |  |  |
| 138 | myofibrom*.tw,kf. | 704 | Advanced |  |  |  |
| 139 | myeloma*.tw,kf. | 56109 | Advanced |  |  |  |
| 140 | myoma*.tw,kf. | 6401 | Advanced |  |  |  |
| 141 | myosarcom*.tw,kf. | 235 | Advanced |  |  |  |
| 142 | myxofibrosarcom*.tw,kf. | 571 | Advanced |  |  |  |
| 143 | myxoma*.tw,kf. | 9437 | Advanced |  |  |  |
| 144 | myxosarcom*.tw,kf. | 263 | Advanced |  |  |  |
| 145 | n?evocarcin*.tw,kf. | 79 | Advanced |  |  |  |
| 146 | neurilemmom*.tw,kf. | 2320 | Advanced |  |  |  |
| 147 | neurocytom*.tw,kf. | 798 | Advanced |  |  |  |
| 148 | neuroectodermal*.tw,kf. | 6909 | Advanced |  |  |  |
| 149 | neurofibroma*.tw,kf. | 18693 | Advanced |  |  |  |
| 150 | neurofibrosarcom*.tw,kf. | 417 | Advanced |  |  |  |
| 151 | neurilemmom*.tw,kf. | 2320 | Advanced |  |  |  |
| 152 | neuroblastom*.tw,kf. | 37570 | Advanced |  |  |  |
| 153 | neuroma*.tw,kf. | 10159 | Advanced |  |  |  |
| 154 | neurothekeom*.tw,kf. | 265 | Advanced |  |  |  |
| 155 | NSCLC?.tw,kf. | 47006 | Advanced |  |  |  |
| 156 | odontoma*.tw,kf. | 1488 | Advanced |  |  |  |
| 157 | oligo*.tw,kf. | 272188 | Advanced |  |  |  |
| 158 | osteoblastom*.tw,kf. | 1216 | Advanced |  |  |  |
| 159 | osteochondrom*.tw,kf. | 3249 | Advanced |  |  |  |
| 160 | osteoclastom*.tw,kf. | 376 | Advanced |  |  |  |
| 161 | osteoma*.tw,kf. | 11412 | Advanced |  |  |  |
| 162 | osteosarcom*.tw,kf. | 24929 | Advanced |  |  |  |
| 163 | papilloma*.tw,kf. | 61688 | Advanced |  |  |  |
| 164 | papillary*.tw,kf. | 58776 | Advanced |  |  |  |
| 165 | paragangliom*.tw,kf. | 7727 | Advanced |  |  |  |
| 166 | pheochromocytom*.tw,kf. | 18126 | Advanced |  |  |  |
| 167 | phyllo?des*.tw,kf. | 2337 | Advanced |  |  |  |
| 168 | pinealocytoma*.tw,kf. | 17 | Advanced |  |  |  |
| 169 | pinealoma*.tw,kf. | 362 | Advanced |  |  |  |
| 170 | pineoblastoma*.tw,kf. | 441 | Advanced |  |  |  |
| 171 | pineocytoma*.tw,kf. | 269 | Advanced |  |  |  |
| 172 | plasmacytom*.tw,kf. | 6815 | Advanced |  |  |  |
| 173 | (polycythem* adj2 vera?).tw,kf. | 5802 | Advanced |  |  |  |
| 174 | prolactinom*.tw,kf. | 3486 | Advanced |  |  |  |
| 175 | retinoblastom*.tw,kf. | 16809 | Advanced |  |  |  |
| 176 | rhabdoid*.tw,kf. | 2832 | Advanced |  |  |  |
| 177 | rhabdomyom*.tw,kf. | 1588 | Advanced |  |  |  |
| 178 | rhabdomyosarcom*.tw,kf. | 12272 | Advanced |  |  |  |
| 179 | sarcom*.tw,kf. | 113723 | Advanced |  |  |  |
| 180 | seminoma*.tw,kf. | 7454 | Advanced |  |  |  |
| 181 | Sertoli- Leydig.tw,kf. | 659 | Advanced |  |  |  |
| 182 | somatostatinoma*.tw,kf. | 393 | Advanced |  |  |  |
| 183 | somatotrophinom*.tw,kf. | 80 | Advanced |  |  |  |
| 184 | struma ovarii*.tw,kf. | 598 | Advanced |  |  |  |
| 185 | thecoma*.tw,kf. | 451 | Advanced |  |  |  |
| 186 | teratocarcin*.tw,kf. | 2682 | Advanced |  |  |  |
| 187 | teratoma*.tw,kf. | 15770 | Advanced |  |  |  |
| 188 | thymom*.tw,kf. | 9634 | Advanced |  |  |  |
| 189 | trophoblast*.tw,kf. | 23104 | Advanced |  |  |  |
| 190 | vipoma*.tw,kf. | 390 | Advanced |  |  |  |
| 191 | wilms*.tw,kf. | 10116 | Advanced |  |  |  |
| 192 | or/1-191 | 5054536 | Advanced |  |  |  |
| 193 | Implementation Science/ | 588 | Advanced |  |  |  |
| 194 | Health Plan Implementation/ | 6225 | Advanced |  |  |  |
| 195 | Health Planning Guidelines/ | 4125 | Advanced |  |  |  |
| 196 | exp Planning Techniques/ | 14499 | Advanced |  |  |  |
| 197 | Strategic Planning/ | 206 | Advanced |  |  |  |
| 198 | Capacity Building/ | 2725 | Advanced |  |  |  |
| 199 | Translational Medical Research/ | 11313 | Advanced |  |  |  |
| 200 | Information Dissemination/ | 17407 | Advanced |  |  |  |
| 201 | Health Information Exchange/ | 926 | Advanced |  |  |  |
| 202 | exp "Diffusion of Innovation"/ | 20299 | Advanced |  |  |  |
| 203 | exp Program Development/ | 29513 | Advanced |  |  |  |
| 204 | exp Program Evaluation/ | 77574 | Advanced |  |  |  |
| 205 | "plan*-act*".tw,kf. | 2924 | Advanced |  |  |  |
| 206 | "plan-do-check".tw,kf. | 220 | Advanced |  |  |  |
| 207 | adapt*.tw,kf. | 574719 | Advanced |  |  |  |
| 208 | deadapt*.tw,kf. | 102 | Advanced |  |  |  |
| 209 | adopt*.tw,kf. | 266097 | Advanced |  |  |  |
| 210 | deadopt*.tw,kf. | 15 | Advanced |  |  |  |
| 211 | implement*.tw,kf. | 532010 | Advanced |  |  |  |
| 212 | deimplement*.tw,kf. | 50 | Advanced |  |  |  |
| 213 | innovat*.tw,kf. | 144237 | Advanced |  |  |  |
| 214 | exnovat*.tw,kf. | 9 | Advanced |  |  |  |
| 215 | disseminat*.tw,kf. | 136132 | Advanced |  |  |  |
| 216 | assimilat*.tw,kf. | 28004 | Advanced |  |  |  |
| 217 | diffus*.tw,kf. | 402629 | Advanced |  |  |  |
| 218 | (knowledge* adj3 translat*).tw,kf. | 6005 | Advanced |  |  |  |
| 219 | (kt adj3 translat*).tw,kf. | 458 | Advanced |  |  |  |
| 220 | (knowledge* adj3 transfer*).tw,kf. | 4108 | Advanced |  |  |  |
| 221 | (kt adj3 transfer*).tw,kf. | 50 | Advanced |  |  |  |
| 222 | (knowledge* adj3 exchang*).tw,kf. | 1478 | Advanced |  |  |  |
| 223 | (kt adj3 exchang*).tw,kf. | 17 | Advanced |  |  |  |
| 224 | (information* adj3 exchang*).tw,kf. | 7947 | Advanced |  |  |  |
| 225 | (plan* adj3 guideline?).tw,kf. | 1225 | Advanced |  |  |  |
| 226 | (plan* adj3 technique?).tw,kf. | 5827 | Advanced |  |  |  |
| 227 | (plan* adj3 strateg*).tw,kf. | 15764 | Advanced |  |  |  |
| 228 | (program* adj3 develop*).tw,kf. | 49871 | Advanced |  |  |  |
| 229 | (program* adj3 evaluat*).tw,kf. | 25904 | Advanced |  |  |  |
| 230 | (capacity adj3 build*).tw,kf. | 8380 | Advanced |  |  |  |
| 231 | (sustain* adj3 change?).tw,kf. | 2111 | Advanced |  |  |  |
| 232 | (sustain* adj3 model*).tw,kf. | 2315 | Advanced |  |  |  |
| 233 | sustainabilit*.tw,kf. | 27094 | Advanced |  |  |  |
| 234 | (quality adj3 chasm).tw,kf. | 252 | Advanced |  |  |  |
| 235 | third mission.tw,kf. | 15 | Advanced |  |  |  |
| 236 | implementation science*.jw. | 1920 | Advanced |  |  |  |
| 237 | or/193-236 | 2153210 | Advanced |  |  |  |
| 238 | Patient Reported Outcome Measures/ | 7382 | Advanced |  |  |  |
| 239 | Patient Outcome Assessment/ | 4933 | Advanced |  |  |  |
| 240 | Patient Health Questionnaire/ | 502 | Advanced |  |  |  |
| 241 | Self Report/ | 34871 | Advanced |  |  |  |
| 242 | (electronic* adj3 patient? adj3 report*).tw,kf. | 407 | Advanced |  |  |  |
| 243 | (electronic* adj3 patient? adj3 (feedback* or feed-back*)).tw,kf. | 12 | Advanced |  |  |  |
| 244 | ((online* or on-line*) adj3 patient? adj3 report*).tw,kf. | 114 | Advanced |  |  |  |
| 245 | ((online* or on-line*) adj3 patient? adj3 (feedback* or feed-back*)).tw,kf. | 32 | Advanced |  |  |  |
| 246 | (internet* adj3 patient? adj3 report*).tw,kf. | 32 | Advanced |  |  |  |
| 247 | (internet* adj3 patient? adj3 (feedback* or feed-back*)).tw,kf. | 4 | Advanced |  |  |  |
| 248 | (computer* adj3 patient? adj3 report*).tw,kf. | 77 | Advanced |  |  |  |
| 249 | (computer* adj3 patient? adj3 (feedback* or feed-back*)).tw,kf. | 4 | Advanced |  |  |  |
| 250 | (digital* adj3 patient? adj3 report*).tw,kf. | 48 | Advanced |  |  |  |
| 251 | (digital* adj3 patient? adj3 (feedback* or feed-back*)).tw,kf. | 2 | Advanced |  |  |  |
| 252 | (web* adj3 patient? adj3 report*).tw,kf. | 85 | Advanced |  |  |  |
| 253 | (web* adj3 patient? adj3 (feedback* or feed-back*)).tw,kf. | 11 | Advanced |  |  |  |
| 254 | (virtual* adj3 patient? adj3 report*).tw,kf. | 40 | Advanced |  |  |  |
| 255 | (virtual* adj3 patient? adj3 (feedback* or feed-back*)).tw,kf. | 3 | Advanced |  |  |  |
| 256 | (remote* adj3 patient? adj3 report*).tw,kf. | 37 | Advanced |  |  |  |
| 257 | (remote* adj3 patient? adj3 (feedback* or feed-back*)).tw,kf. | 3 | Advanced |  |  |  |
| 258 | (tele* adj3 patient? adj3 report*).tw,kf. | 148 | Advanced |  |  |  |
| 259 | (tele* adj3 patient? adj3 (feedback* or feed-back*)).tw,kf. | 12 | Advanced |  |  |  |
| 260 | (mobile* adj3 patient? adj3 report*).tw,kf. | 32 | Advanced |  |  |  |
| 261 | (mobile* adj3 patient? adj3 (feedback* or feed-back*)).tw,kf. | 5 | Advanced |  |  |  |
| 262 | (EHR? adj3 patient? adj3 report*).tw,kf. | 31 | Advanced |  |  |  |
| 263 | (integrat* adj3 patient? adj3 report*).tw,kf. | 143 | Advanced |  |  |  |
| 264 | (electronic* adj3 self* adj3 report*).tw,kf. | 250 | Advanced |  |  |  |
| 265 | (electronic* adj3 self* adj3 (feedback* or feed-back*)).tw,kf. | 5 | Advanced |  |  |  |
| 266 | ((online* or on-line*) adj3 self* adj3 report*).tw,kf. | 664 | Advanced |  |  |  |
| 267 | ((online* or on-line*) adj3 self* adj3 (feedback* or feed-back*)).tw,kf. | 2 | Advanced |  |  |  |
| 268 | (internet* adj3 self* adj3 report*).tw,kf. | 108 | Advanced |  |  |  |
| 269 | (internet* adj3 self* adj3 (feedback* or feed-back*)).tw,kf. | 1 | Advanced |  |  |  |
| 270 | (computer* adj3 self* adj3 report*).tw,kf. | 182 | Advanced |  |  |  |
| 271 | (computer* adj3 self* adj3 (feedback* or feed-back*)).tw,kf. | 2 | Advanced |  |  |  |
| 272 | (digital* adj3 self* adj3 report*).tw,kf. | 32 | Advanced |  |  |  |
| 273 | (digital* adj3 self* adj3 (feedback* or feed-back*)).tw,kf. | 2 | Advanced |  |  |  |
| 274 | (web* adj3 self* adj3 report*).tw,kf. | 198 | Advanced |  |  |  |
| 275 | (web* adj3 self* adj3 (feedback* or feed-back*)).tw,kf. | 1 | Advanced |  |  |  |
| 276 | (virtual* adj3 self* adj3 report*).tw,kf. | 9 | Advanced |  |  |  |
| 277 | (virtual* adj3 self* adj3 (feedback* or feed-back*)).tw,kf. | 0 | Advanced |  |  |  |
| 278 | (remote* adj3 self* adj3 report*).tw,kf. | 22 | Advanced |  |  |  |
| 279 | (remote* adj3 self* adj3 (feedback* or feed-back*)).tw,kf. | 2 | Advanced |  |  |  |
| 280 | (tele* adj3 self* adj3 report*).tw,kf. | 206 | Advanced |  |  |  |
| 281 | (tele* adj3 self* adj3 (feedback* or feed-back*)).tw,kf. | 0 | Advanced |  |  |  |
| 282 | (mobile* adj3 self* adj3 report*).tw,kf. | 60 | Advanced |  |  |  |
| 283 | (mobile* adj3 self* adj3 (feedback* or feed-back*)).tw,kf. | 2 | Advanced |  |  |  |
| 284 | (EHR? adj3 self* adj3 report*).tw,kf. | 19 | Advanced |  |  |  |
| 285 | (EHR? adj3 self* adj3 (feedback* or feed-back*)).tw,kf. | 1 | Advanced |  |  |  |
| 286 | (integrat* adj3 self* adj3 report*).tw,kf. | 76 | Advanced |  |  |  |
| 287 | (integrat* adj3 self* adj3 (feedback* or feed-back*)).tw,kf. | 4 | Advanced |  |  |  |
| 288 | (electronic* adj3 report* adj3 (symptom? or outcome?)).tw,kf. | 305 | Advanced |  |  |  |
| 289 | ((online? or on-line?) adj3 report* adj3 (symptom? or outcome?)).tw,kf. | 77 | Advanced |  |  |  |
| 290 | (internet* adj3 report* adj3 (symptom? or outcome?)).tw,kf. | 10 | Advanced |  |  |  |
| 291 | (computer* adj3 report* adj3 (symptom? or outcome?)).tw,kf. | 43 | Advanced |  |  |  |
| 292 | (digital* adj3 report* adj3 (symptom? or outcome?)).tw,kf. | 27 | Advanced |  |  |  |
| 293 | (web* adj3 report* adj3 (symptom? or outcome?)).tw,kf. | 37 | Advanced |  |  |  |
| 294 | (virtual* adj3 report* adj3 (symptom? or outcome?)).tw,kf. | 13 | Advanced |  |  |  |
| 295 | (remote* adj3 report* adj3 (symptom? or outcome?)).tw,kf. | 19 | Advanced |  |  |  |
| 296 | (tele* adj3 report* adj3 (symptom? or outcome?)).tw,kf. | 38 | Advanced |  |  |  |
| 297 | (mobile* adj3 report* adj3 (symptom? or outcome?)).tw,kf. | 26 | Advanced |  |  |  |
| 298 | (EHR? adj3 report* adj3 (symptom? or outcome?)).tw,kf. | 6 | Advanced |  |  |  |
| 299 | (integrat* adj3 report* adj3 (symptom? or outcome?)).tw,kf. | 105 | Advanced |  |  |  |
| 300 | (electronic* adj3 (PRO? or PROM?)).tw,kf. | 184 | Advanced |  |  |  |
| 301 | ((online? or on-line?) adj3 (PRO? or PROM?)).tw,kf. | 58 | Advanced |  |  |  |
| 302 | (internet* adj3 (PRO? or PROM?)).tw,kf. | 25 | Advanced |  |  |  |
| 303 | (computer* adj3 (PRO? or PROM?)).tw,kf. | 78 | Advanced |  |  |  |
| 304 | (digital* adj3 (PRO? or PROM?)).tw,kf. | 32 | Advanced |  |  |  |
| 305 | (web* adj3 (PRO? or PROM?)).tw,kf. | 196 | Advanced |  |  |  |
| 306 | (virtual* adj3 (PRO? or PROM?)).tw,kf. | 30 | Advanced |  |  |  |
| 307 | (remote* adj3 (PRO? or PROM?)).tw,kf. | 28 | Advanced |  |  |  |
| 308 | (tele* adj3 (PRO? or PROM?)).tw,kf. | 32 | Advanced |  |  |  |
| 309 | (mobile* adj3 (PRO? or PROM?)).tw,kf. | 23 | Advanced |  |  |  |
| 310 | (EHR? adj3 (PRO? or PROM?)).tw,kf. | 10 | Advanced |  |  |  |
| 311 | (integrat* adj3 (PRO? or PROM?)).tw,kf. | 264 | Advanced |  |  |  |
| 312 | ePRO?.tw,kf. | 226 | Advanced |  |  |  |
| 313 | mPRO?.tw,kf. | 488 | Advanced |  |  |  |
| 314 | tPRO?.tw,kf. | 48 | Advanced |  |  |  |
| 315 | ePROM?.tw,kf. | 55 | Advanced |  |  |  |
| 316 | mPROM?.tw,kf. | 2 | Advanced |  |  |  |
| 317 | tPROM?.tw,kf. | 12 | Advanced |  |  |  |
| 318 | eRAPID.tw,kf. | 11 | Advanced |  |  |  |
| 319 | mRAPID.tw,kf. | 0 | Advanced |  |  |  |
| 320 | tRAPID.tw,kf. | 11 | Advanced |  |  |  |
| 321 | PROMIS.tw,kf. | 2131 | Advanced |  |  |  |
| 322 | or/238-321 | 52159 | Advanced |  |  |  |
| 323 | 192 and 237 and 322 | 890 | Advanced |  |  |  |
| 324 | exp animals/ not (exp animals/ and exp humans/) | 4782806 | Advanced |  |  |  |
| 325 | 323 not 324 | 889 | Advanced |  |  |  |
| 326 | limit 325 to "all child (0 to 18 years)" | 162 | Advanced |  |  |  |
| 327 | limit 325 to "all adult (19 plus years)" | 485 | Advanced |  |  |  |
| 328 | 326 not 327 | 79 | Advanced |  |  |  |
| 329 | 325 not 328 | 810 | Advanced |  |  |  |
