# Supplementary material for: Implementation of electronic prospective surveillance models in cancer care: a scoping review
Source: Implement Sci. 2023 Apr 26;18:11. doi: 10.1186/s13012-023-01265-4 (PMC10134630; doi:10.1186/s13012-023-01265-4)
Supplement: Supplementary file 3 — Additional file 3. Adapted ERIC descriptions for implementation strategies used in the included interventions. [file 13012_2023_1265_MOESM3_ESM.docx]

Additional File 3. Adapted ERIC descriptions for implementation strategies used in the included interventions

| ERIC Cluster & Strategy | Description | n (%) | Action Targets | Temporality |
| --- | --- | --- | --- | --- |
| Train and education stakeholders |  |  |  |  |
| Conduct educational meetings | ﻿Held one-on-one or group meetings targeted toward educating different stakeholder groups to teach them about the ePSM. | 25 (54) | Patients and clinical staff | Preparation and implementation |
| Distribute educational materials | ﻿Distributed educational materials in person, by mail, and/or electronically. Materials included educational videos, manuals, brochures, posters, newsletters and postcards. | 20 (43) | Patients and clinical staff | Preparation and implementation |
| Conduct ongoing training | ﻿Planned and conducted training for the ePSM following an initial session. Training included further orientation of the system and the interpretation and use of symptom scores. | 6 (13) | Clinical staff | Preparation and implementation |
| Develop educational materials | ﻿Developed manuals, toolkits, and other supporting materials to make it easier for stakeholders to learn about the ePSM. Materials included educational videos, manuals, brochures, and newsletters. | 2 (4) | Patients and clinical staff | Preparation and implementation |
| Conduct educational outreach visits | ﻿Travelled and met with providers and other staff in their practice settings to educate them about the ePSM. This included case-based interactive sessions and presentations. | 2 (4) | Clinical staff | Preparation, implementation and sustainment |
| Change infrastructure |  |  |  |  |
| Change record systems | ﻿Modified or changed systems to facilitate implementation. This included integrating the ePSM system into the electronic medical record or another existing electronic system, and/or to a patient portal. | 19 (41) | Patients and clinical staff | Preparation |
| Change equipment | ﻿Modified the physical structure and/or equipment to accommodate implementation of the ePSM. This included setting up computer stations or obtaining tablets for patients to complete ePROs in the clinic.﻿ | 9 (20) | Patients | Preparation |
| Engage consumers |  |  |  |  |
| Intervene with patient to enhance adherence | ﻿Developed strategies to support adherence. This included generating system alerts to patients based on inactivity, using in-person reminders to complete ePRO reporting when patients attended clinic, completing the first ePRO report under supervision, or receiving a phone call from staff based on patient inactivity. | 19 (41) | Patients | Implementation and sustainment |
| Involve patients and family members | ﻿Engaged or included patients in the implementation effort. This included using a participatory design with patients to develop the symptom questions. | 4 (9) | Patients | Preparation |
| Prepare patients to be active participants | ﻿Prepared patients to be active in their care and to ask questions. This included coaching patients to verbalize specifics tailored for each symptom to providers such as how often and when the issue occurs, the intensity, and alleviating or aggravating factors. | 1 (2) | Patients | Implementation |
| Develop stakeholder interrelationships |  |  |  |  |
| Use advisory boards and workgroups | ﻿Engaged a group of stakeholders to provide feedback and advice on implementation. This included reviewing system characteristics and content, monitoring progress on implementation, and making decisions and providing feedback on clinic flow, equipment, and implementation strategies. | 7 (15) | Clinical staff, administrative staff, hospital management, researchers, and health informatics professionals | Preparation and implementation |
| Identify and prepare champions | ﻿Prepared individuals to dedicate themselves to supporting and driving implementation. This included individuals who helped design the ePSM for use with the target population, helped bring providers onboard to participate, and worked alongside site implementation teams to facilitate uptake and use of the ePSM for patient management. | 4 (9) | Clinical staff | Preparation, implementation and sustainment |
| Organize clinician implementation team meetings | ﻿Supported a team of clinicians who were implementing the ePSM. This included giving them protected time to reflect on the implementation effort, share lessons learned through collaborative meetings, and discuss practical issues, barriers, and possible solutions. | 4 (9) | Clinical staff | Preparation and implementation |
| Inform local opinion leaders | ﻿Informed and introduced the ePSM to stakeholders identified as opinion leaders in order to influence providers to adopt it. | 2 (4) | Managers and department and division leads | Preparation |
| Involve executive boards | Engaged governing structures in the implementation effort. | 2 (4) | Administrative leaders, boards of directors, and presidents | Sustainment |
| Conduct local consensus discussions | ﻿Engaged local stakeholders in discussions to reach a consensus on the implementation effort. This included a cocreation process to adapt and refine the ePSM for use with patients and determine how to integrate it into the clinic setting. | 1 (2) | Clinical staff, technology provider, and researchers | Preparation |
| Use an implementation advisor | Received guidance and support from an individual who was dedicated to supporting the implementation of the ePSM. This included coaching and leadership on the implementation methodology. | 1 (2) | Not specified | Preparation, implementation and sustainment |
| Use evaluative and iterative strategies |  |  |  |  |
| Assess readiness and identify barriers and facilitators | ﻿Assessed various aspects of an organization to determine its degree of readiness to implement, barriers that may impede implementation, and strengths that can be used in the implementation effort. This included using surveys for organizational readiness, and interviews and focus groups to understand stakeholders’ perspectives. | 5 (11) | Clinical staff, administrative staff, and patients | Preparation |
| Develop and organize quality monitoring systems | ﻿Developed systems and procedures to monitor clinical processes and/or outcomes for the purpose of quality improvement. This included screening rates, time to complete screening, and reasons for missed screening, as well as the use of rapid plan-do-study-act cycles for improvement. | 3 (7) | Clinical staff | Implementation |
| Audit and provide feedback | ﻿Collected and summarized clinical performance data over a specified time period and provided this to clinicians and administrators to monitor, evaluate, and modify behaviour. This included ePRO completion rates, proportion of patients meeting clinical thresholds, and changes in symptom scores. | 3 (7) | Clinical staff | Implementation and sustainment |
| Conduct local needs assessment | Evaluated the setting for use of the ePSM. This included an assessment of the setting’s technology infrastructure and human and material resource requirements. | 1 (2) | Clinical staff and researchers | Preparation |
| Provide interactive assistance |  |  |  |  |
| Provide local technical assistance | ﻿Developed and used a system to deliver technical assistance focused on implementation issues. This included local staff assisting patient in setting up their smartphones for ePSM use and setting up a helpdesk service for patients and providers. | 8 (17) | Patients and clinical staff | Implementation and sustainment |
| Support clinicians |  |  |  |  |
| Facilitate relay of clinical data to providers | ﻿Provided as close to real-time data as possible about key measures of process/outcomes using integrated channels of communication in a way that promotes use of the ePSM. This may include an individual, team, or system that transfers symptom alert data from a clinical app/web-app into the clinic’s electronic medical record. | 1 (2) | Clinical staff | Implementation |
| Remind clinicians | Developed a reminder system designed to help clinicians recall information and/or prompt them to use the ePSM. This may include an individual or system that communicates to the appropriate clinician that there is new data to review. | 1 (2) | Clinical staff | Implementation |
| Create new clinical teams | ﻿Made changes to the clinical team, adding different disciplines and different skills to make it more likely that the ePSM was delivered. This included hiring a new secretary to oversee volunteers and patient adherence to the ePROs. | 1 (2) | Administrative staff | Preparation |
| Utilize financial strategies |  |  |  |  |
| Use other payment schemes | ﻿Introduced payment approaches. This included full reimbursement via a health insurance company if the ePSM was offered by a health care provider. | 1 (2) | Patients | Preparation |

ERIC, Expert Recommendations for Implementing Change; ePSM, electronic prospective surveillance model; ePRO, electronic patient-reported outcome. Descriptions are adapted from Powell et al.[22]. n (%), signifies the frequency and percentages of the 46 ePSM interventions included in the review
