# Supplementary material for: Implementation of electronic prospective surveillance models in cancer care: a scoping review
Source: Implement Sci. 2023 Apr 26;18:11. doi: 10.1186/s13012-023-01265-4 (PMC10134630; doi:10.1186/s13012-023-01265-4)
Supplement: Supplementary file 4 — Additional file 4. Adapted descriptions for implementation outcomes reported by the included interventions. [file 13012_2023_1265_MOESM4_ESM.docx]

Additional File 4. Adapted descriptions for implementation outcomes reported by the included interventions

| Outcome | Operationalized Description | n (%) |
| --- | --- | --- |
| Feasibility | - The extent to which the ePSM can be developed to administer ePROs, visualize results, and/or provide recommendations to patients and clinicians in a meaningful way - The length of time it takes patients to complete ePRO reporting - Technical issues and dropouts | 33 (72) |
| Acceptability | - Stakeholder perspectives on whether the ePSM was helpful in managing symptoms, its ease of use, and whether they would recommend the system to others | 31 (67) |
| Appropriateness | - The fit of the ePSM with the patient population (e.g., relevance and meaningfulness of ePROs and self-management material) and clinic values - Perceived relative advantage of the ePSM versus usual care | 18 (39) |
| Fidelity | - Consistency of ePROs completed by patients - Consistency of clinicians who review scores with patients - Adaptations and modifications to the ePSM or implementation | 18 (39) |
| Penetration | - Proportion of patients who registered during a specified period - Rate of missing ePRO data during a specified period | 16 (35) |
| Cost | - Financial impact of the ePSM including staff and time required to administer the intervention and its implementation | 4 (9) |
| Adoption | - The proportion and representativeness of clinics or centres willing to implement the ePSM | 2 (4) |
| Sustainability | - The extent to which the ePSM becomes embedded into the setting’s workflow and policies | 1 (2) |

Descriptions are adapted from Stover et al. [24] ePRO, electronic patient-reported outcome; ePSM, electronic prospective surveillance model. n (%), signifies the frequency and percentages of the 46 ePSM interventions included in the review.
