# Supplementary material for: Implementation of electronic prospective surveillance models in cancer care: a scoping review
Source: Implement Sci. 2023 Apr 26;18:11. doi: 10.1186/s13012-023-01265-4 (PMC10134630; doi:10.1186/s13012-023-01265-4)
Supplement: Supplementary file 5 — Additional file 5. Description of electronic prospective surveillance model system features. [file 13012_2023_1265_MOESM5_ESM.docx]

Additional File 5. Description of electronic prospective surveillance model system features

| **System Feature** | **Description** | **n (%)** |
| --- | --- | --- |
| Patients |  |  |
| Self-Management | Automatically generates information/advice on symptom management including behaviours, practices, or techniques that patients can perform to address specific symptoms or quality of life. May also include the ability to access a library of resources/information at any time to address cancer-related impairments. | 17 (37) |
| Score History | View a report or graph of their own previous results. | 10 (22) |
| System Message | Informs the patient that their scores are not or may not be monitored. States that a potentially serious symptom has been reported and that medical evaluation should be considered. Advises patient when and who to contact if further support is required. | 9 (20) |
| Communication | Ability to message providers or administrators to ask questions or request an e-consult, as well as view messages and instructions from providers. | 4 (9) |
| General Education | Information about tests, treatments, and potential side effects, and/or information about patients' legal rights. | 3 (7) |
| Circle of Care | View the list of attending physicians, their specialties, schedules, and contact information. | 2 (4) |
| Peer Support | Share experiences with other patients by participating in an online forum group discussion that allows them to exchange messages anonymously with other patients or use a blog. | 1 (2) |
| Providers |  |  |
| Summary Report | Provides information on patient symptoms and tracking of scores available through the electronic medical record or on a separate website. May include graphs, colours for thresholds and symptom severity, and analytics (patient use of system). | 41 (89) |
| Alerts | The provider receives an email, message within the electronic medical record or separate system, or alert on a mobile phone designated for the intervention when a patient scores above a certain threshold. | 15 (33) |
| Recommended Action | Provides information on symptom management pathways including recommended clinical actions and suggested referrals. | 5 (11) |
| Communication | Ability to send messages to patients, such as reminders, advice, instructions, prescriptions, and appointment schedules. | 3 (7) |
| Patient List | Ability to view a list of their patients and access their individual records such as demographic data, clinical data, other attending physicians. | 1 (2) |

n (%), signifies the frequency and percentages of the 46 ePSM interventions included in the review
