# Supplementary material for: Implementation of electronic prospective surveillance models in cancer care: a scoping review
Source: Implement Sci. 2023 Apr 26;18:11. doi: 10.1186/s13012-023-01265-4 (PMC10134630; doi:10.1186/s13012-023-01265-4)
Supplement: Supplementary file 6 — Additional file 6. Clusters and discrete implementation strategies used in the included interventions. [file 13012_2023_1265_MOESM6_ESM.docx]

Additional File 6. Clusters and discrete implementation strategies used in the included interventions

|  | **Train and Education Stakeholders** | | | | | **Change Infrastructure** | | **Engage Consumers** | | | **Develop stakeholder interrelationships** | | | | | | | **Use evaluative and iterative strategies** | | | | **Provide interactive assistance** | **Support clinicians** | | |
| --- | --- | --- | --- | --- | --- | --- | --- | --- | --- | --- | --- | --- | --- | --- | --- | --- | --- | --- | --- | --- | --- | --- | --- | --- | --- |
|  | Conduct educational meetings | Distribute educational materials | Conduct ongoing training | Develop educational materials | Conduct educational outreach visits | Change record systems | Change equipment | Intervene to enhance adherence | Involve patients and family members | Prepare patients to be active participants | Use advisory boards | Prepare champions | Clinician implementation team meetings | Inform local opinion leaders | Involve executive boards | Conduct local consensus discussions | Use an implementation advisor | Assess readiness | Organize quality monitoring systems | Audit and provide feedback | Conduct local needs assessment | Provide local technical assistance | Facilitate relay of clinical data to providers | Remind clinicians | Create new clinical teams |
| Abernethy 2009 [64] |  | ✓ |  |  |  |  | ✓ | ✓ |  |  |  |  |  |  |  |  |  |  |  |  |  |  |  |  |  |
| Absolom 2021 [27,75,76] |  |  | ✓ | ✓ |  | ✓ |  |  | ✓ |  | ✓ |  |  |  |  |  |  |  |  |  |  |  |  |  |  |
| Bacorro 2018 [70] |  |  |  | ✓ |  | ✓ |  |  |  |  |  |  |  |  |  |  |  |  |  |  |  |  |  |  |  |
| Baeksted 2017 [29] | ✓ |  |  |  |  | ✓ |  |  |  |  |  |  |  |  |  |  |  |  | ✓ |  |  |  |  |  |  |
| Basch 2005 [57] | ✓ | ✓ |  |  |  |  | ✓ | ✓ |  |  |  |  |  |  |  |  |  |  |  |  |  |  |  |  |  |
| Basch 2007 [56] | ✓ |  |  |  |  |  | ✓ | ✓ |  |  |  |  |  |  |  |  |  |  |  |  |  |  |  |  |  |
| Basch 2016 [14] | ✓ |  |  |  |  |  |  |  |  |  |  |  |  |  |  |  |  |  |  |  |  |  |  |  |  |
| Basch 2020 [54] | ✓ |  |  |  |  |  |  | ✓ |  |  |  |  |  |  |  |  |  |  |  |  |  |  |  |  |  |
| Berry 2014 [15,77] | ✓ |  |  |  |  |  |  | ✓ |  | ✓ |  |  |  |  |  |  |  |  |  |  |  |  |  |  |  |
| Biran 2020 [50] | ✓ | ✓ |  |  |  |  |  |  | ✓ |  |  |  |  |  |  | ✓ |  |  |  |  |  |  | ✓ | ✓ |  |
| Børøsund 2014 [24] | ✓ | ✓ |  |  |  |  |  |  |  |  |  |  |  |  |  |  |  |  |  |  |  | ✓ |  |  |  |
| Cowan 2016 [49,78] | ✓ |  |  |  |  |  |  | ✓ |  |  |  |  |  |  |  |  |  |  |  |  |  |  |  |  |  |
| de Wit 2019 [30] | ✓ | ✓ |  |  |  |  |  |  | ✓ |  | ✓ |  |  |  |  |  |  | ✓ |  |  |  | ✓ |  |  |  |
| Dronkers 2020 [31] | ✓ |  |  |  |  | ✓ | ✓ | ✓ |  |  |  |  |  |  |  |  |  |  |  |  |  |  |  |  | ✓ |
| Dudgeon 2012 [63,79,80] |  |  |  | ✓ | ✓ |  | ✓ |  |  |  | ✓ | ✓ | ✓ |  | ✓ |  | ✓ |  | ✓ |  |  |  |  |  |  |
| Duman-Lubberding 2017 [32] |  |  |  |  |  | ✓ | ✓ |  |  |  |  |  |  |  |  |  |  |  |  |  |  |  |  |  |  |
| Erharter 2010 [33] |  | ✓ | ✓ |  |  |  |  |  |  |  |  |  |  |  |  |  |  |  |  |  |  |  |  |  |  |
| Fernandez 2019 [34] |  | ✓ | ✓ |  |  |  |  |  |  |  |  |  | ✓ |  |  |  |  |  |  |  |  | ✓ |  |  |  |
| Garcia 2019 [52] |  |  |  |  |  | ✓ |  |  |  |  |  |  |  |  |  |  |  |  |  |  |  |  |  |  |  |
| Girgis 2017 [68,81] | ✓ | ✓ |  |  |  | ✓ |  | ✓ |  |  | ✓ |  |  |  |  |  |  |  |  |  |  |  |  |  |  |
| Girgis 2020 [67,82] |  |  |  |  |  | ✓ |  | ✓ |  |  |  |  |  |  |  |  |  |  |  |  |  |  |  |  |  |
| Hackett 2020 [35,83] | ✓ | ✓ |  |  |  |  |  |  |  |  |  |  |  |  |  |  |  |  |  |  |  |  |  |  |  |
| Hansen 2021 [36,72] | ✓ | ✓ |  |  |  | ✓ |  |  |  |  |  |  |  |  |  |  |  |  |  |  |  |  |  |  |  |
| Hauth 2019 [37] |  |  |  |  |  |  |  | ✓ |  |  |  |  |  |  |  |  |  |  |  |  |  |  |  |  |  |
| Howell 2020 [51, 74] | ✓ | ✓ | ✓ |  | ✓ | ✓ |  |  |  |  |  | ✓ | ✓ | ✓ | ✓ |  |  | ✓ |  | ✓ |  |  |  |  |  |
| Kneuertz 2020 [59] |  |  |  |  |  |  |  |  |  |  |  |  |  |  |  |  |  |  |  |  |  |  |  |  |  |
| Li 2016 [60] | ✓ | ✓ |  |  |  | ✓ | ✓ | ✓ |  |  | ✓ |  |  | ✓ |  |  |  | ✓ | ✓ | ✓ |  |  |  |  |  |
| Maguire 2015 [39] | ✓ |  |  |  |  |  |  |  | ✓ |  | ✓ |  |  |  |  |  |  |  |  |  |  |  |  |  |  |
| Maguire 2020 [38] |  |  |  |  |  |  |  |  | ✓ |  |  |  |  |  |  |  | ✓ |  |  |  |  |  |  |  |  |
| Maguire 2021 [40,84,85] | ✓ | ✓ |  |  |  |  |  |  |  |  |  |  | ✓ |  |  |  |  | ✓ |  |  | ✓ |  |  |  |  |
| Mark 2008 [66] |  |  |  |  |  |  |  | ✓ |  |  |  |  |  |  |  |  |  |  |  |  |  |  |  |  |  |
| Mouillet 2021 [41] |  | ✓ | ✓ |  |  |  |  |  |  |  |  |  |  |  |  |  |  |  |  |  |  |  |  |  |  |
| Naughton 2020 [55] |  |  |  |  |  | ✓ |  | ✓ |  |  |  | ✓ |  |  |  |  |  |  |  |  |  | ✓ |  |  |  |
| Riis 2021 [42] | ✓ |  |  |  |  |  |  |  |  |  |  |  |  |  |  |  |  |  |  |  |  |  |  |  |  |
| Roberts 2020 [69,73] | ✓ | ✓ |  |  |  | ✓ |  |  |  |  |  | ✓ |  |  |  |  |  | ✓ |  | ✓ |  | ✓ |  |  |  |
| Rotenstein 2017 [61] | ✓ | ✓ |  |  |  | ✓ | ✓ |  |  |  |  |  |  |  |  |  |  |  |  |  |  |  |  |  |  |
| Strachna 2021 [53] |  |  |  |  |  | ✓ |  | ✓ |  |  |  |  |  |  |  |  |  |  |  |  |  |  |  |  |  |
| Sundberg 2017 [43] | ✓ |  |  |  |  |  |  | ✓ |  |  |  |  |  |  |  |  |  |  |  |  |  | ✓ |  |  |  |
| Taarnhoj 2020 [44] |  | ✓ | ✓ |  |  | ✓ |  |  |  |  |  |  |  |  |  |  |  |  |  |  |  | ✓ |  |  |  |
| Tolstrup [45,71] | ✓ |  |  |  |  |  |  |  |  |  |  |  |  |  |  |  |  |  |  |  |  |  |  |  |  |
| van der Hout 2020 [46] |  |  |  |  |  |  |  |  |  |  |  |  |  |  |  |  |  |  |  |  |  | ✓ |  |  |  |
| van Eenbergen 2019 [47] | ✓ | ✓ |  |  |  |  |  |  |  |  |  |  |  |  |  |  |  |  |  |  |  |  |  |  |  |
| Wagner 2015 [58] |  |  |  |  |  | ✓ |  | ✓ |  |  |  |  |  |  |  |  |  |  |  |  |  |  |  |  |  |
| Wu 2016 [62,86] | ✓ | ✓ |  |  |  | ✓ |  | ✓ |  |  |  |  |  |  |  |  |  |  |  |  |  |  |  |  |  |
| Zebralla 2020 [48] |  |  |  |  |  |  | ✓ |  |  |  |  |  |  |  |  |  |  |  |  |  |  |  |  |  |  |
| Zylla 2020 [65] |  |  |  |  |  | ✓ |  | ✓ |  |  |  |  |  |  |  |  |  |  |  |  |  |  |  |  |  |

de Wit 2019 [30] utilized an additional strategy: Use other payment schemes
